# An Extracellular Matrix Aging Clock Based on Circulating Matrisome Proteins Predicts Biological Aging and Disease

**DOI:** 10.1111/acel.70474

**Published:** 2026-04-15

**Authors:** Loet Coenen, Adrian Molière, Benoit Lehallier, Jinte Middeldorp, Collin Y. Ewald

**Affiliations:** ^1^ Department of Neurobiology & Aging Biomedical Primate Research Centre Rijswijk the Netherlands; ^2^ Department of Molecular Cell Biology and Immunology Amsterdam Neuroscience, Amsterdam UMC Location Vrije Universiteit Amsterdam Amsterdam the Netherlands; ^3^ Laboratory of Extracellular Matrix Regeneration, Institute of Translational Medicine, Department of Health Sciences and Technology ETH Zürich Schwerzenbach Switzerland; ^4^ Alkahest Inc San Carlos California USA

**Keywords:** aging, biomarkers, extracellular matrix proteins, proteomics

## Abstract

Plasma proteomic aging clocks estimate biological age and are linked to age‐related diseases, but thus far, there has been little focus on circulating extracellular matrix (ECM) proteins, which play a central role in tissue structure and age‐related decline. Here, we use publicly available proteomic datasets to profile plasma ECM protein abundances across the human lifespan and reveal a distinct U‐shaped trajectory with age. Our ECM‐based aging clock, constructed from 14 plasma proteins, accurately predicts chronological age and remains robust across independent validations and different biofluids. Notably, this ECM clock distinguishes between healthy and diseased states. Cross‐species comparisons show that while specific predictive ECM proteins differ between humans and rodents, species‐specific ECM clocks reliably track aging, and rejuvenation interventions can reverse ECM aging signatures. We prioritize ECM proteins implicated causally in disease and explored drugs targeting these proteins. These findings establish circulating ECM proteins as sensitive biomarkers of aging and disease and suggest that targeting ECM remodeling may offer new strategies for promoting healthy aging.

## Graphical abstract

A 14‐protein extracellular matrix aging clock derived from circulating matrisome proteins predicts chronological and biological age across cohorts and biofluids, distinguishes health from disease, and is modulated by rejuvenation interventions in mice, enabling the nomination of potential ECM targets for drug discovery to promote healthy aging.

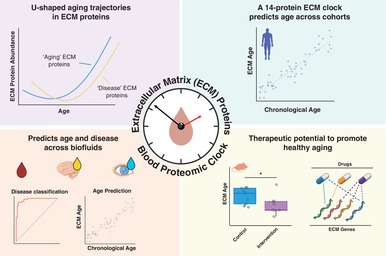

## 1 Introduction

Aging manifests through complex physiological changes across multiple biological scales and contributes to the development of the majority of diseases, necessitating robust biomarkers for quantifying physiological and pathological age‐related decline (Rutledge et al. 2022). An accumulation of high‐throughput data across various omics levels has led to the development of various aging clocks, machine learning models trained to predict the chronological age of samples (Biomarkers of Aging Consortium et al. 2024; Horvath 2013; Jung et al. 2023; Meer et al. 2018; Petkovich et al. 2017; Ying et al. 2024). In this context, the predicted age is often interpreted as more reflective of the actual biological age of the individual, as it is based on age‐associated biological changes (Biomarkers of Aging Consortium et al. 2024). Recent advances in proteomic profiling have revealed that circulating blood proteins serve as robust predictive markers of chronological age and age‐associated pathologies, offering unprecedented insights into systemic and organ‐specific deterioration (Biomarkers of Aging Consortium et al. 2024; Deng et al. 2025; Kivimaki et al. 2025; Oh et al. 2023).

The extracellular matrix (ECM) is the non‐cellular component of tissues in which all cells are embedded (Frantz et al. 2010). Beyond its well‐characterized role in providing structural support and acting as a scaffold for cell motility, the ECM also plays crucial roles in various cellular functions through the regulation of intercellular communication and the distribution of signaling molecules (Hynes 2009; J. Y. C. Park et al. 2023). Thus, cell and tissue function cannot be fully understood independently of the ECM.

The subset of the proteome made up of ECM‐related proteins is called the matrisome (Naba et al. 2016). There are a total of 1027 proteins making up the human matrisome. The matrisome can be further divided into the core‐ and associated matrisome. The core matrisome is made up of collagens (44 proteins), glycoproteins (195 proteins), and proteoglycans (35 proteins). These proteins are synthesized and secreted by cells directly to build the ECM. The associated matrisome entails secreted factors (344 proteins), ECM regulators (238 proteins), and ECM‐affiliated proteins (171 proteins). The associated matrisome is defined as proteins that localize at or remodel the ECM.

The matrisome encompasses all ECM proteins that can be expressed; however, ECM composition and modification are specific to cell and tissue types and change differentially during various biological states, for example, during disease development. To disentangle various states of the ECM associated with or caused by certain conditions or phenotypes, the concept of “matreotypes” has been established, defined as a snapshot of the composition and modification of matrisome proteins associated with a specific phenotype (Ewald 2020).

The matrisome has been shown to associate strongly with the aging process (Coenen et al. 2023; Lehallier et al. 2020), undergoing a series of progressive changes characterized by distinct matreotypes. These changes include oxidation, glycation, crosslinking, collagen fragmentation, and protein aggregate accumulation (Ewald 2020) and are implicated in the loss of physiological function and the development of age‐related diseases (Bonnans et al. 2014; Frantz et al. 2010). However, how the matrisome changes during aging and how this may contribute to disease susceptibility remains unclear.

Furthermore, specific aging phenotypes can be reversed simply by relocating aged cells and diseased tissue to young ECM, as seen with aged stem cells (Sun et al. 2011), or by systemic injection of youthful ECM remodeling factors (Castellano et al. 2017), highlighting the crucial role of the ECM in determining cellular phenotypes and disease development and illustrating its therapeutic potential. These considerations make plasma matrisome proteins prime candidates for assessing physiological health and diseased states.

Here, we characterize the changes in circulating ECM protein abundances with age using publicly available SomaScan datasets. We show that ECM proteins have U‐shaped trajectories with age and are more strongly associated with disease than other proteins. Matrisome protein abundances in the plasma can be used to build accurate aging clocks that perform comparably to unbiased aging clocks and are applicable across datasets and different biofluids. Moreover, we provide evidence that heterochronic parabiosis rejuvenates the ECM in a mouse model and put forward several druggable matrisome proteins that are putative causal candidates in a range of human diseases, highlighting the matrisome proteins as potential therapeutic targets to improve the quality of the aging process.

## 2 Results

### 2.1 Age‐Associated Changes of ECM Proteins in the Plasma Proteome

**FIGURE 1:**
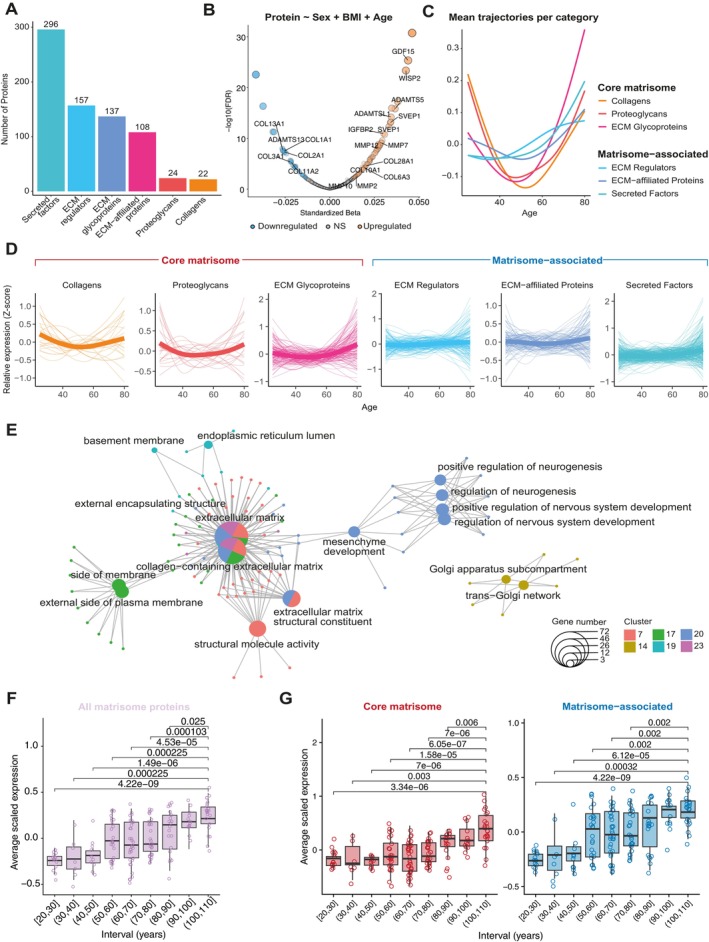
Plasma ECM proteins showed distinct U‐shaped trajectories with age. (A) Overview of the number of matrisome proteins (MPs) per matrisome category in the dataset of Arthur et al. 2021. (B) Results of the linear modeling in Arthur et al. (2021) performed on standardized protein abundances, with the specified model on top. *x*‐axis denotes the effect size and *y*‐axis denotes the −log10(FDR) value. Labeled proteins are highlighted based on functional description in the text. Sizes and opacity of datapoints are scaled to the estimated effect sizes. (C) Relative expression trajectories of *z*‐scored protein abundances across samples per category of MP over time. (D) Zoom in on a single category of MPs, including its underlying proteins. Thick lines reflect the mean expression trajectory, and thin lines denote *z*‐scored protein abundances across the trajectories of individual proteins. (E) Cnetplot denoting the enriched function/process in the bigger circles, with the connected proteins in each small circle. Colors denote in which cluster the function or protein is found. (F, G) average standardized abundance levels of the 382 matrisome proteins across different age groups from the Lehallier et al. (2019) dataset. Comparisons highlighted by the brackets are between the given group and the centenarian group (100–110 years) and are accompanied by FDR‐adjusted *p*‐values obtained using Wilcoxon tests.

To better understand how changes in the ECM during aging are reflected in the plasma proteome, we used two public datasets of two independent cohorts providing plasma proteomic measurements using the SomaScan platform of 150 individuals aged 25–80 years (Arthur et al. 2021) and of 736 individuals aged 16–66 years (Robbins et al. 2021). Despite cohort differences ‒0 for example, sex distributions, used anticoagulant, and ethnic backgrounds ‐ both cohorts contained data on around 740 aptamers targeting any of the 1027 human matrisome proteins (Dataset Arthur = 744 aptamers, Dataset Robbins = 740 aptamers). The 744 matrisome proteins detected in the Arthur dataset consisted of approximately 300 secreted factors, 160 ECM regulators, 130 ECM glycoproteins, 110 ECM‐affiliated proteins, 25 proteoglycans, and 22 collagens in plasma (Figure 1A). This represents an overall coverage of 50%–75% of the known matrisome in human blood.

We next re‐analyzed the dataset of Arthur et al. (2021) as they provide the largest age range, and replicated the analyses in Robbins et al. (2021) (Figure S1A). We first determined which matrisome protein levels in the blood were associated with aging. Using linear models, out of the 740 matrisome proteins, we identified significant (FDR < 0.05) positive associations with age for 112 and 118 proteins and negative association for 47 and 103 matrisome proteins for the Arthur et al. (2021) and Robbins et al. (2021) cohorts, respectively (Figures 1B and S1B, Tables S1 and S4). Associations with age across cohorts were significant (Pearson's *r* = 0.561, *p* = 2.961e‐62), suggesting robust associations with age. We found both positive and negative associations with age across both datasets for collagens (e.g., COL1A1, COL11A2, COL18A1) that form the ECM and a higher abundance of enzymes that remodel the ECM (i.e., ADAMTS5, ADAMTSL1, MMP7), consistent with the idea of ECM turnover during aging (Figures 1B and S1B). The most substantial increases during aging were seen within secreted factors, including GDF15, IGFBP2, WISP2, and SVEP1 (Figures 1B and S1B, Tables S1 and S4).

Previous work has established non‐linear abundance trajectories of plasma proteins during aging (Coenen et al. 2023; Lehallier et al. 2020; Shen et al. 2024). Therefore, we investigated matrisome protein abundance levels in the plasma cross‐sectionally over time. When combining aging trajectories of core matrisome and matrisome‐associated proteins, ECM regulators and ECM‐affiliated proteins showed minimal changes in abundance trajectory, with indications of moderately increased levels across ECM‐associated proteins from 50 to 60 years onward. (Figures 1C and S1C). This contrasts with the core matrisome proteins, showing U‐shaped trajectories with the lowest point preceding these changes around the age of 40–50 years (Figures 1C and S1D). Similarly, secreted factors showed an increase after the age of 60 years (Figures 1C and S1D). However, underlying proteins driving this signal showed considerable heterogeneity in their aging trajectories (Figure 1D). Given that the rise of secreted factors, collagens, proteoglycans, and ECM glycoproteins starts around 50–60 years, the U‐shaped trajectories were less pronounced yet still visible in the Robbins et al. (2021) cohort. This is expected as the oldest individual in the Robbins dataset is 66 years old (Figure S1C,D). Overall, these findings point to extensive remodeling of the ECM during aging, characterized by non‐linear abundance changes.

We further hypothesized that the senescence‐associated secretory phenotype (SASP) of accumulating senescent cells during aging may have driven this increase in ECM changes and secreted factors. Secreted factors are stored in the ECM, and their U‐shaped trajectory suggested that remodeling of the ECM could lead to the release of these factors (Basisty et al. 2020), including proteins from different senescence inducers, cell types, and exosomes. More than half of these proteins (991 SASP proteins) were detected in the Arthur dataset, of which 170 were matrisome proteins (SASP‐ECM proteins). However, the complete SASP signature in the plasma remained on average relatively stable during aging, with some SASPs increasing after the age of 70 years, and we observed no temporal trajectory co‐occurring with the observed U or J‐shaped trajectories of ECM and matrisome secreted factors (Figure S2).

To identify the processes that accompany or potentially induce changes in ECM protein levels in the blood during aging, we applied an unbiased clustering approach based on the relative protein abundance trajectories of the complete measured proteome. We identified 8 clusters significantly enriched for ECM proteins in the Arthur dataset (FDR < 0.05; Table S2) and 9 for the Robbins dataset (FDR < 0.05; Table S5). Across the two cohorts, we observed significant enrichments for processes involved in ECM degradation and de novo synthesis (e.g., peptidase regulator activity, external encapsulating structures, Golgi and endoplasmic reticulum structures, collagen chain trimerization) and processes either involved in development or disease transition, such as neuronal and glial differentiation, bone morphogenesis, epithelial‐mesenchymal transition, and diseases of glycosylation (Figures 1E and S1E, Tables S3 and S6).

One limitation of the previously used datasets is that they are relatively sparse in very old age. To investigate ECM protein dynamics in very old age, we used the dataset of Lehallier et al. (2019). Although this dataset provides a lower resolution on the plasma proteome with 1305 proteins (of which 382 matrisome proteins) compared to the Arthur and Robbins datasets, this dataset included data on a broader age range of 171 healthy aging individuals, including the unique age group of 23 centenarians (overall age range: 21–107 years).

When comparing the matrisome protein abundance levels across age groups, we did not identify significant differences in protein abundance levels of all matrisome proteins in individuals from 90 years onwards compared to centenarians (Figure 1F), suggesting relatively younger profiles in the older age groups. This pattern was likely driven by matrisome‐associated proteins, which appeared to plateau after 80 years. Although no significant differences were found between 90 and 100‐year‐old individuals and centenarians for the core matrisome proteins as well, an increasing trend was still observed (Figure 1G). Together, these findings may suggest that centenarians have an ECM plasma proteomic profile fitting with individuals at least 10 years younger, which could indicate a decelerated aging process.

### 2.2 ECM Dynamics in Disease Associated With Unique Plasma Proteomic Signatures

The ECM has been shown to undergo extensive remodeling in a diseased state (J. Y. C. Park et al. 2023). Given the observed U‐shaped curve in aging, one hypothesis is that after development and reproduction, ECM remodeling declines as a consequence of aging, coinciding with the curve's low point between 40 and 50 years, but then ECM remodeling increases again between the ages of 60–70 years due to the development of age‐related diseases. Thus, two signatures (aging and disease) might overlap to form this U‐shaped curve. To disentangle this, the concept of matreotypes is useful. A matreotype is a snapshot of the ECM composition associated with or caused by a phenotype, including aging and disease status (Ewald 2020). Therefore, we postulated that the combination of aging‐matreotypes and disease‐matreotypes underlied the observed U‐shaped associations.

**FIGURE 2:**
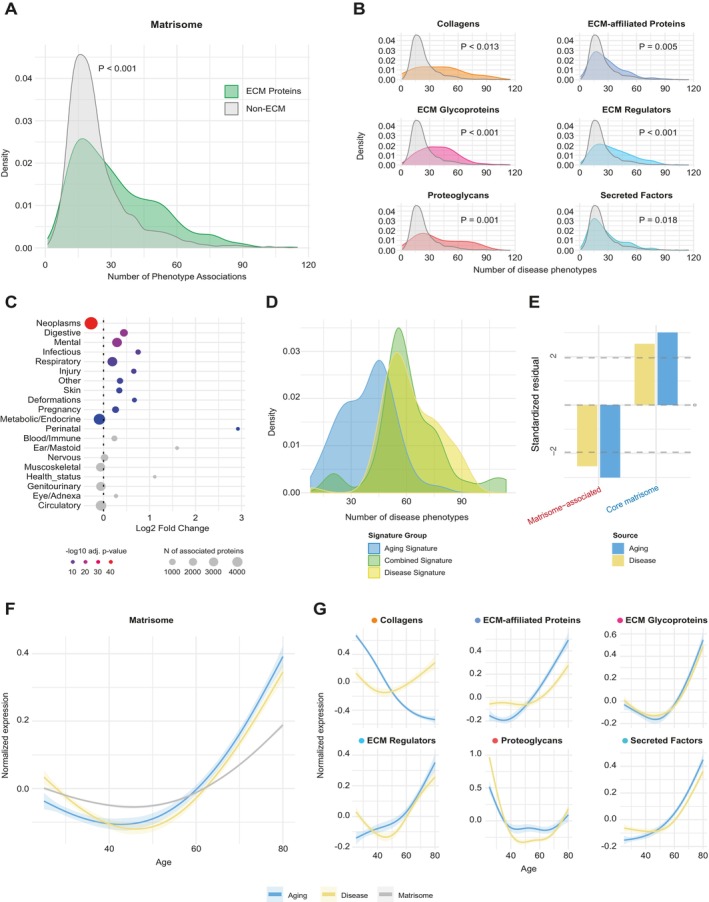
Age‐related changes in ECM proteins were strongly associated with disease. (A, B) Density of disease associations of all MPs (A) and individual matrisome categories (B) based on the number of phenotypes each protein is associated with. Significance was calculated using the unpaired Wilcoxon test followed by Bonferroni correction. (C) Enrichment or depletion of ICD10 disease chapter‐associated phenotypes for ECM proteins compared to all non‐ECM proteins. Shown on the *x*‐axis is the log2 enrichment of disease chapters in ECM proteins versus non‐ECM proteins. The color indicates −log10‐transformed Bonferroni‐adjusted *p*‐values; gray color indicates *p* > 0.05. (D) Phenotype associations for either ECM proteins associated exclusively with aging, disease, or those shared in both signatures. Comparison of Aging and Disease signature: *p* < 0.001. Determined using the Welch two‐sample *t*‐test. (E) Standardized residuals from chi‐squared goodness‐of‐fit tests comparing the distribution of the core and associated matrisome in Disease (gold) and Aging (blue) protein signatures against the expected distribution from the full matrisome proteome. Positive residuals indicate over‐representation (enrichment) and negative residuals indicate under‐representation (depletion) of specific categories. Vertical dashed lines mark statistical significance thresholds at ±1.96 (*p* < 0.05). (F) Smoothed and normalized mean protein expression trajectories are shown as a function of chronological age in the Arthur dataset (blue: Aging signature, gold: Disease signature, gray: All matrisome proteins). 95% confidence intervals are indicated for each line. (G) Similar to (F), but stratified by ECM category (ECM glycoproteins, collagens, proteoglycans, ECM‐affiliated proteins, ECM regulators, and secreted factors).

To test this idea, we used a public dataset providing protein‐phenotype associations using the SomaScan platform of ~35,000 Icelandic individuals (Eldjarn et al. 2023). Across 162 included disease phenotypes, 4583 plasma proteins were significantly associated with at least one phenotype after multiple testing corrections. Of all matrisome proteins, 677 were associated with at least one of the disease phenotypes (disease‐matreotypes) after correction for age and sex effects. Interestingly, we found that, on average, matrisome proteins were associated with more disease phenotypes (mean = 32.2 diseases) than non‐ECM proteins (mean = 23.8 diseases; Wilcoxon test, *p* < 0.001; Figure 2A). This difference in the number of disease associations appeared more substantial in the subcategories collagens, ECM glycoproteins, proteoglycans, and ECM regulators (Figure 2B), underlining the role of ECM remodeling in a diseased state.

As the different matrisome components have distinct functions in the ECM, we wondered whether the different components were similarly affected across various diseases. To test this, we first manually classified the disease phenotypes based on the ICD10 classification system (World Health Organization 2019) into 20 disease categories, such as mental and behavioral disorders (e.g., dementia, schizophrenia, mental retardation) and diseases of the respiratory system (e.g., respiratory infections, influenza, pneumonia) (Table S7). Using this approach, we were able to extract plasma protein lists associated with each unique disease category. We found that half of the disease categories were enriched in matrisome proteins after Bonferroni correction, with the most substantial enrichment in mental and digestive diseases (Figure 2C). The top disease‐associated ECM‐related proteins for mental diseases included PLXNB2, SEMA6B, CILP2, ADAMTSL2, and OMD; for digestive diseases, INHBC, LEP, IGFBP1, and IL1RN. On the other hand, two disease categories were significantly depleted in matrisome proteins, namely neoplasms and diseases of metabolism and the endocrine system (Figure 2C). To further understand which matrisome components were most associated with specific disease categories, we examined the individual contributions of each matrisome category to the disease associations. We found that ECM glycoproteins and regulators were significantly associated with most disease categories, while secreted factors showed the least association (Figure S3), matching the overall lower association with disease phenotypes (Figure 2B).

As aging coincides with increased susceptibility to disease, we then tested if the plasma proteomic signature of the aging‐matreotypes differs from the disease‐matreotypes. In short, proteins associated with the disease‐matreotype were defined as those most associated with several diseases across distinct disease categories (see methods Section 5.7). With this method, we derived 93 ECM proteins that were mainly affected across different diseased states (Figure S4, Table S8). We then compared the disease‐matreotype signature to our aging‐matreotype signature. For our aging‐matreotype signature, we included matrisome proteins that were previously associated with age in our analyses (FDR < 0.05) across both datasets with shared directionality (Table S9). This aging‐matreotype signature consisted of 100 proteins. When comparing the aging and disease ECM signatures, we found that 31 proteins are shared between the signatures. As expected, the ECM disease signature was associated with more disease phenotypes than the ECM aging signature (Figure 2D).

To understand if the matrisome was affected functionally in a diseased and aged state, we tested if the core and associated matrisome were affected differently across matreotypes. Overall, there was a depletion in matrisome‐associated proteins and an enrichment for core matrisome proteins (collagens, ECM glycoproteins, proteoglycans) in both aging and disease signatures (Figure 2E) compared to the full matrisome, suggesting that the core matrisome is more affected in both states. On a category level, there was a depletion in secreted factors for both the aging and disease signatures (Figure S5). Moreover, we found that the aging signature was enriched for glycoproteins while the disease signature was enriched for collagens, with the other in both cases trending in the same direction. Interestingly, there were two categories for which aging and disease signatures behaved differently from each other, namely, ECM regulators, which trended towards enrichment for the disease signature, while there was no difference in the aging signature, and proteoglycans, which trended towards an increase in aging and not in disease (Figure S5). This could indicate increased active remodeling of the ECM in a diseased state and loss of crucial ECM components during aging.

To further disentangle this, we asked whether the ECM proteins associated with either aging or disease differ in their abundance trajectories with age. Both sets of proteins showed a more pronounced increase in expression with age compared to all matrisome proteins in Arthur and Robbins (Figures 2F and Figure S6A). Across all categories, the disease signature appears slightly delayed compared to the aging signature, suggesting that age‐associated ECM changes precede or contribute to disease‐associated changes. Looking at the individual ECM categories, we similarly found a delay in changes in disease signature, except for collagens, with the selected collagens in the aging signature being negatively associated with age (Figures 2G and S6B).

Taken together, we found that ECM proteins, in particular those in the core matrisome, are strongly associated with a range of diseases. Furthermore, the changes in the aging‐matreotype slightly preceded changes in the disease‐associated matreotype.

### 2.3 Changes in the Plasma Matrisome Can Predict Chronological Age

As we found strong associations between matrisome proteins and age (aging matreotype), we next questioned whether (1) matrisome protein abundance is predictive of chronological age, (2) how this measure compares to unbiased aging predictions, and (3) whether the ECM age predicts similar biological age differences as unbiased aging predictors.

**FIGURE 3:**
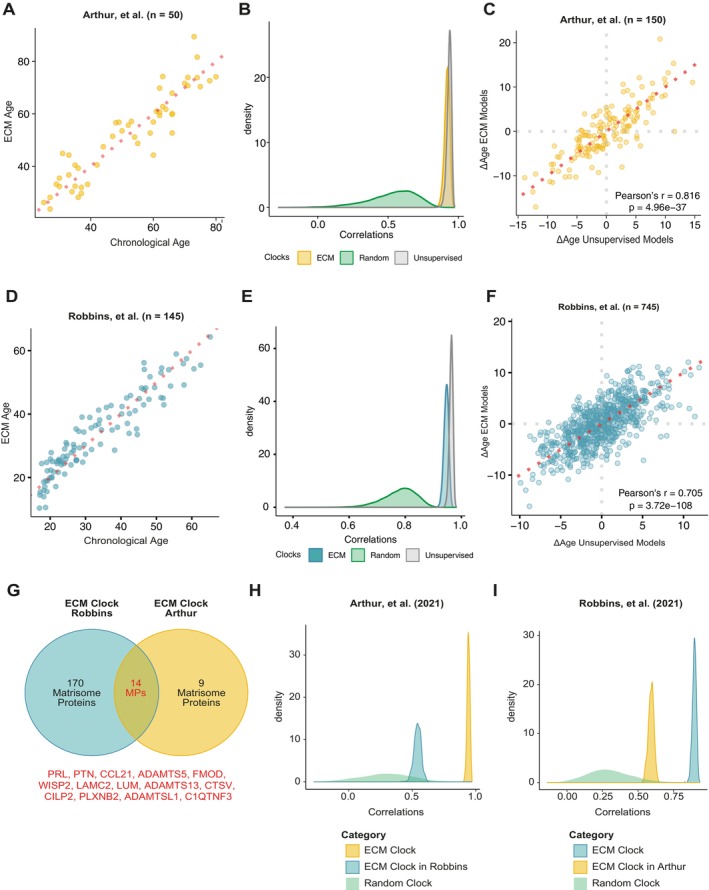
Proteomic clocks based on ECM proteins were reliable predictors of chronological age across cohorts. (A) Example of the application of the ECM Clock model in the dataset of Arthur et al. (B) Distributions of 10,000 correlations found between the predicted and chronological age for the ECM clocks (yellow), unsupervised protein clocks (gray), and random clocks with similar sizes as the ECM clocks (green). (C) Overview of the age gaps found using the ECM clocks (*y*‐axis) and the unsupervised clocks (*x*‐axis). Panel (D–F) represents the same as (A–C) and is derived from the dataset of Robbins et al. (G) Overview of overlapping matrisome proteins (MPs), which are selected across all models. In red below are the selected proteins that overlap in both datasets. (H) Overview of distributions of correlations between the ECM clocks and chronological ages using the 14 selected MPs in the same dataset (green), in the other dataset (blue or yellow), or using the Random clocks (gray). Same for panel (I).

To answer these questions, we created ECM clocks using LASSO regression in randomly permuted subsets of training and testing data using only the matrisome protein abundance and sex as input (for a full description, see the methods Section 5.9). Across all models, we identified a strong positive correlation (*r* = 0.919; Figure 3A) between the average ECM Age and the average Chronological Age (CA). Across all 10,000 ECM Clocks, 23 matrisome proteins were selected in more than half of these models, highlighting these proteins as key players in ECM aging (Table S10). No significant enrichment of a specific matrisome category was found within these selected proteins (all *p* > 0.15). Most of these variables were secreted factors (PTN, CCL21, PRL, IL19, GDF10, IFNA7, MSTN, and GDF3), followed by ECM regulators (ADAMTSL1, CTSV, ADAMTS5, ADAMTS13, TIMP4, and CST6), ECM glycoproteins (CILP2, WISP2, EMILIN3, and LAMC2), ECM‐affiliated proteins (C1QTNF3 and PLXNB2) and proteoglycan (LUM and FMOD), and a collagen (COL10A1).

Unsupervised clocks built without constraints in selecting proteins may capture a broader, unbiased age‐associated signature, while random clocks with a constraint in the number of allowed predictors allow for testing the predictive validity of directed clocks, such as our ECM clock. Thus, we tested whether ECM clocks are equally predictive as unsupervised aging clocks and if they outperform similarly sized random clocks. Using a similar approach to the ECM clocks, we created unsupervised and random clocks to predict CA. Using these unsupervised clocks, we found, on average, a comparable but slightly stronger correlation between predicted and CA (*r* = 0.934; Figure 3B). However, this stronger predictive value came at the cost of more proteins in most of these models (26 proteins). Moreover, we identified significant enrichment of matrisome proteins among these 26 proteins (9 proteins; *p* = 0.007; Table S11), reflecting the importance of the matrisome proteins in aging. Moreover, both clocks strongly outperformed random clocks (*r* = 0.49; Figure 3B).

To further test whether the ECM age reflects similar biological age differences as unbiased aging predictors, we compared the age gaps (the gap between the Chronological Age and Predicted Age, often associated with the quality of aging and risk of age‐associated diseases (Biomarkers of Aging Consortium et al. 2024)) across both clocks. We identified strong positive correlations between the age gaps estimated across the two types of clocks (*r* = 0.817, *p* = 3.75e‐37; Figure 3C), suggesting that the plasma ECM clock functions well as a proxy of broad biological aging.

To explore if these results are translatable in an independent dataset, we reapplied our workflow to the second dataset of Robbins et al. (2021). In line with our initial findings, the ECM clocks were comparable but slightly less predictive in this dataset (*r* = 0.946; Figure 3D) than the unsupervised clocks (*r* = 0.963; Figure 3E), which both outperformed the random clocks (*r* = 0.779; Figure 3E). Although these models showed stronger associations between CA and predicted age across clocks than in our previous dataset, they appeared less stable, with 183 proteins selected in most of the ECM models and 184 in the unsupervised clocks (Tables S12 and S13). Moreover, a strong positive correlation was found between the age gaps of these two clocks (*r* = 0.716, *p* = 1.78e‐111; Figure 3F), again highlighting the predictive value of ECM plasma clocks in the aging process.

Across both datasets, we found an overlap of 14 matrisome proteins that were selected in most of the models (PRL, PTN, CCL21, ADAMTS5, FMOD, WISP2, LAMC2, LUM, ADAMTS13, CTSV, CILP2, PLXNB2, ADAMTSL1, C1QTNF3; Figure 3G). Given their putatively important role in biological age prediction, we then tested (1) if they would outperform the same set of randomly selected proteins within the same dataset and (2) how predictive the models created in one dataset were in predicting the age in the second dataset. In both datasets, the ECM clock using the 14 matrisome proteins outperformed the similarly sized set of random proteins, which was more prominent in the dataset of Arthur (*r*_ECM_ = 0.942, *r*_random_ = 0.292; Figure 3H) than in Robbins (*r*_ECM_ = 0.897, *r*_random_ = 0.297; Figure 3I). Furthermore, we found moderate associations between ECM age predicted using a model trained on an external dataset (average correlations of the Arthur model in Robbins dataset, *r* = 0.541, Robbins model in Arthur dataset, *r* = 0.592), which on average greatly outperformed the random sets of proteins within these datasets (Figure 3I). Together, these results showed that biological age prediction based on matreotype profiles was a good proxy for broad, unbiased biological age predictions and that key matrisome proteins reflect necessary age‐associated signals.

### 2.4 The Preserved ECM Plasma Signature as a Biomarker for Diseased States Across Biomaterials

The blood proteome comprises proteins or protein fragments from different sources, such as secreted proteins from different organs, cell leakage, and cleaved extracellular and membrane proteins. Furthermore, it is subject to changes in a diseased state, and we described earlier that this appears especially in matrisome proteins. Seven of the 14 robust age‐associated matrisome proteins overlapped in our matreotype‐disease signature, and they show predictive abilities across datasets in estimating ages. Together, this led to the question of whether the signature of 14 matrisome proteins is a robust measure of aging throughout the body and if they have further potential as a biomarker for diseases.

**FIGURE 4:**
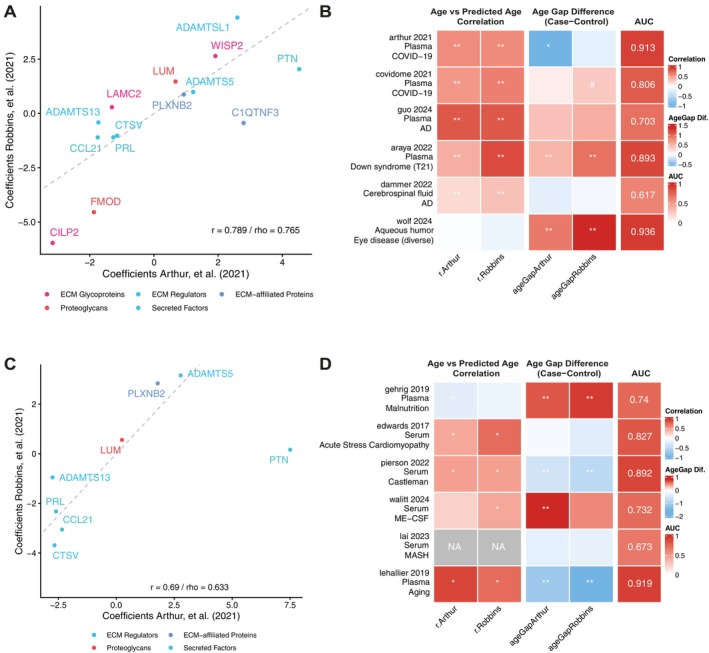
ECM Clocks trained across datasets were good predictors of biological age and disease classifications across diseases and biofluids. (A) Overview of the coefficients by the models trained in the two datasets using 14 proteins. Text color denotes the category of matrisome protein to which they belong, as highlighted in Figure 1. (B) Application of the 14 ECM protein models to external datasets. Correlational strength represents Spearman correlations. Age gap difference and correspondence significance level are estimated by the Wilcoxon tests, with the ‘control’ groups as reference. AUC values indicate the aggregated AUC of the model in this dataset using a leave‐one‐out cross‐validation. **p < 0.01, *p < 0.05, #*p* < 0.10. (C) Overview of the coefficients by the models trained in the two datasets using 8 ECM proteins. Text color denotes the category of matrisome protein to which they belong, as highlighted in Figure 1. (D) Application of the 8 ECM protein models to external datasets. Description of visualized data is similar to that in panel (B).

To test this, we established stable ECM clocks using the 14 matrisome proteins trained across both datasets. The coefficients of these trained models can be found in Table S14. As expected, a correlation between predicted age and chronological age was strong within each dataset and outperformed similar‐sized sets of random proteins (*r*_Arthur_ = 0.955, *r*_Robbins_ = 0.90; Figure S7A,B). Moreover, coefficients across both trained models reflected a high degree of similarity across datasets; however, differences across models are observed (Figure 4A). For example, the protein C1QTNF3 showed a positive coefficient for the model trained in the Arthur et al. dataset and a negative coefficient in the Robbins et al. dataset. This may be caused by the heterogeneity across both datasets (i.e., age range, anti‐coagulant, ethnic composition) reflected in these final models, which should be considered when applying these models in other datasets to obtain reliable results.

We further validated these clocks in external datasets among a range of diseases and biofluids. For a complete overview of included datasets and study summaries, see Table S15. In general, we observed strong correlations between ECM age and chronological age in studies focusing on plasma (Figure 4B). This predictive value remained significant in the cerebrospinal fluid but not in aqueous humor. Moreover, in most studies, the predicted ECM age (‘Age Gap’) appeared higher in a diseased state; however, it only reached significance in a few studies (Figure 4B). Classifiers built using these 14 matrisome proteins revealed great to excellent predictive qualities in distinguishing cases from controls in blood and aqueous humor (AUC range: 0.70–0.94). The lowest area under the curve was observed in the CSF dataset (AUC = 0.617), which may reflect different disease dynamics or altered composition in this biofluid for these proteins. Of note, the significant age gap in the study of Dammer et al. (2022) (Dammer et al. 2022) also correlated significantly with important clinical metadata in Alzheimer's disease (Figure S8), such as amyloid beta and phosphorylated tau levels. This may suggest that the ECM age gap not only distinguishes healthy from disease, but also reflects disease severity.

As we found limited datasets containing information on all 14 matrisome proteins, we further explored if reduced models trained with data on a previous version of the SomaScan platform (1.3k) remained predictive. In this platform, 8 out of 14 matrisome proteins were still measured. The coefficients of these models trained across the two datasets can be found in Table S16. Correlation between predicted age and chronological age was strong in the dataset of Arthur et al. (2021) (*r*_Age_ = 0.88) but less strong in the model trained in the dataset of Robbins et al. (2021) (*r*_Age_ = 0.614). Across both datasets, these predictors still outperformed similarly sized sets of random proteins (Figure S7C,D). Coefficients across both trained models again reflected a large degree of similarity across datasets (Figure 4C).

Surprisingly, when using these reduced models with only approximately half of the previously selected matrisome proteins, moderate correlations between ECM age and chronological age persisted across biomaterials and a variety of diseases (Figure 4D). Furthermore, we still observed several higher age gaps in cases versus controls, with the notable exception of centenarians having a lower biological age in the dataset of Lehallier et al. (2019), further supporting our earlier findings (Figure 1F). Although it should be noted that a lower prediction in centenarians could also be driven by a regression to the mean, as those ages lie outside the trained age range. The reduced models remained predictive in distinguishing cases and controls in the diseases tested (AUC range: 0.67–0.92). Applying the reduced clocks to our previous datasets (Figure 4B) showed highly similar results. Although the effects appear less pronounced, likely due to the inclusion of fewer ECM proteins, this further supports the age‐associated signatures of these proteins and validates our earlier results (Figure S7E). Altogether, these results revealed 14 matrisome proteins tightly associated with aging and disease across diseases and biofluids.

### 2.5 Predictive ECM Clocks in Humans and Mice Suggest Distinct Drivers of Matrisome Aging and Reveal Therapeutic Opportunities in Mice

Most species and all mammals show signs of aging (Cohen 2018; Finch 2009; Nussey et al. 2013). However, to date, it remains an open question which processes are conserved and if there are common biomarkers for these shared processes. Mice are important model systems in aging research; however, the extent to which ECM aging is shared across species remains unclear. To investigate if the aging matrisome signature in the plasma was conserved across species, we reused the dataset of Lehallier et al. (2019), which also includes data on 110 mice (age range: 1–30 months). Of these mice, 29 were used in a heterochronic parabiosis experiment, a well‐described procedure to study the systemic rejuvenating effects of young plasma (Conboy et al. 2013; Zhang et al. 2023).

**FIGURE 5:**
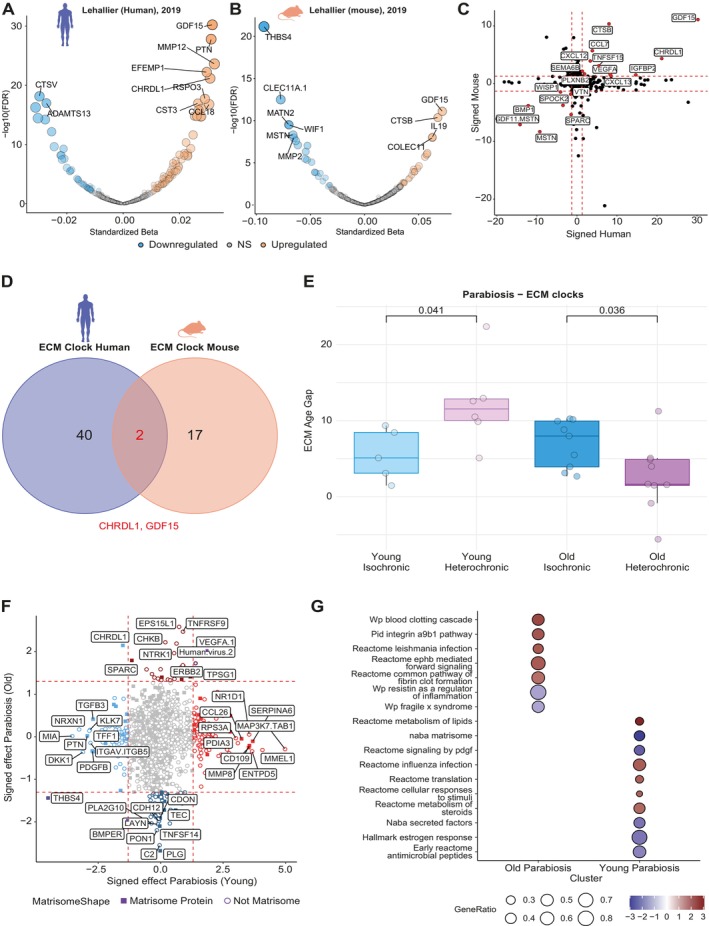
Humans and mice showed conserved matrisome aging with distinct proteomic drivers. (A) Results of linear models in the human dataset of Lehallier et al. (2019). (B) Results of linear models in the mouse dataset in Lehallier et al. (C) Labeled proteins are MPs showing a similar age‐ and effect direction in the plasma across mouse and humans (*p* < 0.05). Signed effects are computed by the sign of the effect (− or +)*−log10(*p*‐value). (D) Venn diagram depicting the overlapping proteins between the ECM Clocks in mice and the ECM Clocks in humans. (E) Results of the ECM age prediction for the parabiosis cohort in Lehallier et al. Comparisons are only made between the groups, with the brackets denoting the *p*‐value obtained from Welch's *t*‐test. (F) Comparison of associations between circulating protein levels and ECM age gap across young and old parabiosis cohorts. Points highlighted in red show positive associations with age gaps (‘accelerated aging’) and in blue show negative associations with age gaps (‘rejuvenation’). Lighter colors reflect the young group and darker colors the old group. Labeled points are the top 10 proteins within each quadrant. Purple points are proteins showing the same effects across groups. A square shape denotes whether the protein is part of the matrisome. (G) Top 5 strongest up‐ and downregulated gene sets based on gene set enrichment analysis across parabiotic age groups.

We first tested if the matrisome proteins in this dataset (382 matrisome proteins) showed similar aging effects in the plasma proteome across species. In humans, we identified 118 matrisome proteins to be associated with aging (FDR < 0.05; Figure 5A, Table S17), in which the majority showed, in line with our previous results, a positive association (77 proteins). For mice, we identified a significant association with age for 48 matrisome proteins (FDR < 0.05; Table S18), of which 25 proteins showed a positive association with age (Figure 5B). Estimated age effects for all matrisome proteins did not correlate between humans and mice (*r* = 0.0385, *p* = 0.453). Human age effects did correlate with the estimated effects of Arthur et al. (*r* = 0.663) and Robbins et al. (*r* = 0.422), whereas mouse did not (*r*_Arthur_ = 0.061; *r*_Robbins_ = 0.119). Of all matrisome proteins, 18 showed similar age associations between species (*p* < 0.05 and similar association with age), of which nine proteins were secreted factors (CCL7, CHRDL1, CXCL12, CXCL13, GDF11, GDF15, MSTN, TNFSF15, and VEGFA), four ECM Glycoproteins (IGFBP2, SPARC, VTN, and WISP1), two ECM regulators (BMP1 and CTSB), and two ECM‐affiliated Proteins (PLXNB2 and SEMA6B), and one proteoglycan (SPOCK2; Figure 5C).

Next, we recreated both ECM and random aging clocks for both species as described earlier (see methods Section 5.9) to obtain more reliable measures of ECM aging, as this dataset measures fewer proteins. For the Human Clocks, of all 14 proteins in our presented stable clock, 8 were measured in the dataset of Lehallier et al. (2019). Of these proteins, 7 proteins (ADAMTS13, ADAMTS5, CCL21, CTSV, LUM, PLXNB2, PTN) were again included in most of the independently created models for ECM age prediction in humans in this dataset (Figure 5D; Table S20). In line with previous findings, correlations between chronological and predicted age were marginally stronger in the Unsupervised clocks (*r* = 0.910) compared to ECM clocks (*r* = 0.897), and biological ages across both clocks remained similar (*r* = 0.864, *p* = 2.269e‐52). Again, a trend towards enrichment of matrisome proteins was observed in the most selected variables of the Unsupervised clocks (*p* = 0.086; Table S21).

For the Mouse Clocks, only control animals not subjected to parabiosis were used to train the models. We identified stronger correlations for ECM age clocks (mean *r* = 0.945) than random clocks (mean *r* = 0.934). Moreover, fewer proteins were used in the majority of the models for the ECM Clocks (19 proteins; Table S22) compared to the Unsupervised models (22 proteins). Similar to the human models, we identified strong correlations in the biological ages across the two approaches (*r* = 0.906, *p* = 4.670e‐42) and observed an enrichment of matrisome proteins among the most selected proteins in the Unsupervised Clocks (*p* = 0.01; Table S23). When comparing the Human‐ECM clock to the Mouse‐ECM clock, it revealed only two secreted factors in overlap (CHRDL1, GDF15; Figure 5D). These results suggest that alterations in the matrisome appear conserved across species; however, the proteins driving these changes may differ across species. It should be noted that a human‐based platform was used, and variability in probe binding across species could also contribute to a lack of shared protein signatures.

Heterochronic parabiosis experiments can show both rejuvenating and aging effects throughout the body (Bieri et al. 2023). Using data from these experiments, we tested whether heterochronic parabiosis may also lead to alterations in the aging or ECM signature, as reflected in a smaller or negative (‘rejuvenating’) age gap (‘ΔAge’) based on these clocks. To do so, we applied the clocks we had developed in the aging mouse dataset to the parabiotic mice. For the ECM clocks, we identified a significantly higher ECM age gap in Young Heterochronic (YH) mice (ΔAge = 11.54) compared to Young Isochronic (YI) mice (ΔAge = 5.09; *p* = 0.041; Figure 5E). Moreover, we identified a lower age gap in the Old Heterochronic (OH) mice (ΔAge = 2.60) compared to the Old Isochronic (OI) mice (ΔAge = 6.92; *p* = 0.036; Figure 5E). When applying the Unsupervised clocks, we find similar results as when applying the ECM clocks, with an increased gap for YH compared to YI (ΔAge = 8.04 and 0.77, respectively; *p* = 0.01) and an increased age gap for OI compared to OH (ΔAge = 4.05 and −0.65, respectively; *p* = 4.72e‐03; Figure S9). In line with our previous findings, these lower age gaps compared to the ECM clocks suggest that the Unsupervised models appear to be better predictors for chronological age than solely the matrisome proteins. Interestingly, the ECM models predict higher age gaps than the Unsupervised models across all parabiosis groups. This suggests that the parabiosis‐induced stress is associated with ECM proteins specifically.

We further investigated which proteins and their functional associations may drive accelerated aging or rejuvenation of the ECM. To do so, we applied a linear model within the Old and Young parabiosis groups separately (i.e., OI and OH combined, and YI parabionts grouped with YH) to associate protein abundance levels with the observed age gap, while correcting for the effects explained by the parabiotic intervention (isochronic or heterochronic). We observed distinct proteomic drivers of rejuvenation and age acceleration across both groups (Figure 5F, Table S23), although these results did not reach significance after correction for multiple testing. Only two proteins showed similar effects in terms of accelerated (VEGFA) or decelerated ECM aging (THBS4) across age groups.

Gene set enrichment analysis further suggests distinct mechanisms underlying accelerated aging or rejuvenation of the ECM across age groups (Figure 5G, Table S24). Among the top hits (nominal *p* < 0.05) in the old parabionts, we observed diverse gene sets associated with ECM aging, among which an inflammatory signal (e.g., integrin A9B1 pathway, Leishmania infection, Resistin as regulator of inflammation) was present. In the young parabiosis group, we observed several gene sets associated with metabolism (e.g., metabolism of lipids, steroids, and protein translation) and inflammatory response (e.g., influenza infection, antimicrobial peptides) to associate with age gaps. Rejuvenating responses were linked to genes associated with PDGF and estrogen signaling. Together, this may indicate that parabiosis may have a body‐wide rejuvenating effect in mice, which is in part reflected in the matrisome, but also affects the matrisome. Importantly, we observed rejuvenating effects for matrisome proteins and secreted factors, highlighting the importance and therapeutic possibilities of the matrisome in aging and rejuvenation.

### 2.6 Genetically Informed Matrisome Protein Prioritization Reveals Important Druggable Targets

**FIGURE 6:**
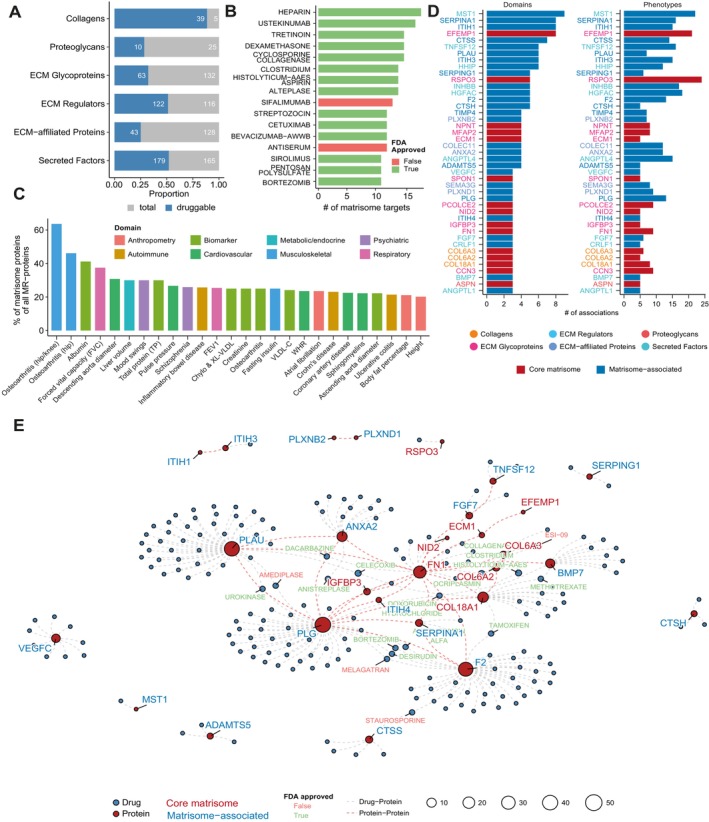
Drug repurposing opportunities targeting prioritized matrisome proteins. (A) Proportion of druggable matrisome proteins (dark blue) across each of the matrisome proteins (gray) in the different categories. Numbers represent the absolute number of proteins. (B) Overview of the number of matrisome targets for each drug. Green color represents an FDA‐approved drug, red color denotes a non‐FDA‐approved drug. (C) Overview of the relevant involvement of matrisome proteins among all causally implied proteins. Colors denote different domains corresponding to the phenotypes. (D) Overview of individual protein associations across different domains and phenotypes. Texted color denotes the matrisome category of the protein. Color in bars reflects if the protein is part of the core matrisome (red) or matrisome‐associated (blue). (E) Interaction network of proteins among each other (red lines, StringDB interaction score > 700) and proteins with drugs (gray lines). Proteins are shown as red nodes, and their corresponding color denotes if it is part of the core—(orange) or associated with the matrisome (blue). Highlighted drug nodes reflect FDA‐approved drugs (green) or other drugs (red) that influence at least two prioritized proteins. The size of the nodes depicts the number of connections. Abbreviations in panel (C): Chylo & XL‐VLDL = Concentration of chylomicrons and extremely large VLDL, FEV1 = Forced expiratory volume in 1‐s, VLDL‐C = Very low‐density lipoprotein cholesterol.

To further explore the option of the matrisome as a therapeutic option in humans, we first examined which matrisome proteins are known drug targets. Using the Human Protein Atlas (Jiang et al. 2020) and Drug Gene Interaction database (Cannon et al. 2024), we found that 461 matrisome proteins are druggable, of which 298 are already targeted by FDA‐approved compounds. These drugs predominantly act on matrisome‐associated factors (Figure 6A), and several top‐ranking compounds have been previously suggested as potential geroprotective agents (i.e., Sirolimus, Aspirin; Figure 6B).

Following the geroscience hypothesis, stating that targeting key biological pathways of aging may provide an efficient therapeutic option to combat multiple age‐related diseases, we mapped which matrisome proteins influenced multiple disease domains. Using the results of a phenome‐wide Mendelian randomization study across 355 phenotypes and 22 disease domains (Su et al. 2024), we observed the highest involvement (> 20%) of matrisome proteins in musculoskeletal phenotypes, specifically osteoarthritis (Figure 6C). Other substantial associations were found for circulating biomarkers often used in the clinic (e.g., albumin, total protein, lipid measures) and both structural (i.e., aorta diameter) and functional (i.e., pulse pressure) cardiovascular traits.

We further prioritized candidate matrisome proteins implicated in at least five phenotypes across at least three domains, leaving us with 43 proteins, including 15 from the core matrisome (Figure 6D). Top‐ranking proteins included MST1, SERPINA1, ITIH1, EFEMP1, and CTSS, which are involved in at least 7 domains and more than 10 phenotypes. Of all prioritized candidates, approximately half are druggable targets (21/43 proteins), of which 16 have FDA‐approved drugs. Only SPON1 overlapped between our disease‐ and aging‐matreotypes, while 12 proteins were part of our aging matreotype (ADAMTS5, ASPN, EFEMP1, HHIP, INHBB, ITIH1, ITIH3, MFAP2, NPNT, PLXNB2, SEMA3G, and TIMP4), and 6 were part of our disease matreotype (ANGPTL1, COL18A1, COL6A3, ECM1, RSPO3, and TNFSF12).

We then explored the interaction landscape among these prioritized proteins and drugs. Using the String database (Szklarczyk et al. 2023), we observed a strong interconnected network centered around Fibronectin 1 (FN1) and Plasminogen (PLG) (Figure 6E), with additional highly connected proteins including PLAU, ANXA2, F2, SERPINA1, COL18A1, and COL6A2. Among 191 drugs targeting these proteins, 16 targeted more than one prioritized target, the majority of which were FDA‐approved. The FDA‐approved drugs Ocriplasmin, Celecoxib, and Collagenase *Clostridium histolyticum* ‐aaes showed the broadest targeting across matrisome proteins. Together, these results underscore the involvement of the matrisome in disease and explore its therapeutic potential to promote healthy aging.

## 3 Discussion

Despite the crucial role of the ECM in aging, an in‐depth characterization of ECM protein changes with age is lacking. Here, we set out to systematically characterize ECM protein dynamics using large‐scale SomaScan data (Arthur et al. 2021; Eldjarn et al. 2023; Lehallier et al. 2019; Robbins et al. 2021). We characterized their abundance trajectories with age and across species, investigated their disease association, built accurate predictors of age and diseased state, and investigated how druggable disease‐related ECM proteins are.

We found that between 20% and 30% of matrisome proteins are associated with age, with the majority being positive associations (Figure 1B). While linear changes with age have been extensively characterized, non‐linear dynamics remain comparatively understudied. Recent research, however, has revealed widespread non‐linear changes across multiple omics layers (Shen et al. 2024). In line with this, the trajectories of core matrisome proteins were U‐shaped, with secreted factors showing a J‐shaped increase (Figure 1C,D). The observed U‐shaped trajectory suggests that extracellular matrix (ECM) remodeling occurs both during development and again during aging, beginning around 50 years of age. It has been proposed that the dysregulation and partial reactivation of developmental programs may contribute to the biological aging process (Ermolaeva et al. 2018; Kern 2023; Martin et al. 2014). Supporting this notion, recent molecular evidence indicates that developmental genes bound by Polycomb Repressive Complex 2 (PRC2) become epigenetically derepressed with age (Moqri et al. 2024). Our finding of U‐shaped ECM protein trajectories may thus reflect a re‐engagement of developmental processes during aging. Such a reengagement could be the result of active remodeling of the ECM during aging, either in the form of pathological misexpression caused by improper signaling not optimized for optimal function in a deteriorating soma, or compensatory mechanisms employed to counteract age‐related deleterious changes (Kern et al. 2024; Syed et al. 2025). Another possibility is stochastic damage, which has been proposed recently to drive aging, particularly in the epigenome (Meyer and Schumacher 2024; Tarkhov et al. 2024; Tong et al. 2024). Indeed, epigenetic clocks can be built solely based on the accumulation of stochastic variation (Meyer and Schumacher 2024). Under such a framework, impaired epigenetic regulatory fidelity caused by stochastic damage leads to the pathological de‐repression of ECM remodeling‐related proteins and pathways. Detangling these possible drivers will be a major task for future research.

Previous work has demonstrated that the blood proteome can be used to build accurate biological clocks (Argentieri et al. 2024; Biomarkers of Aging Consortium et al. 2024; Lehallier et al. 2020). Despite the often non‐linear trajectories of ECM proteins, we were able to develop accurate chronological age predictors using only plasma ECM proteins. These ECM‐based clocks performed comparably to models trained on the full proteome, underscoring the central role of the ECM in the aging process (Figure 3). Fourteen matrisome proteins were shared across the majority of clocks in both datasets, namely: PRL, PTN, COL21, ADAMTS5, FMOD, WISP2, LAMC2, LUM, ADAMTS13, CTSV, CILP2, PLXNB2, ADAMTSL1, and C1QTNF3. Half of the 14 proteins are also in the disease signature (FMOD, WISP2, ADAMTS13, CTSV, CILP2, ADAMTSL1, C1QTNF3). Many of the ECM clock proteins we found have previously been associated with age‐related diseases in the literature, especially vascular disease and atherosclerosis (ADAMTS5 (Z. Wang et al. 2019), LUM (Luo et al. 2024), ADAMTS13 (Bongers et al. 2009), CTSV (Li et al. 2019), CILP2 (Hu et al. 2020)) as well as bone‐related diseases and osteoarthritis (ADAMTS5 (Glasson et al. 2005), FMOD (Li et al. 2019), LUM (S. J. Park et al. 2024), WISP2 (Ruiz‐Fernandez et al. 2022; Tanaka et al. 2005), CILP2 (Bernardo et al. 2011)) and various cancers (PRL (Bessette et al. 2008), CTSV (Xia et al. 2022), LAMC2 (Fu et al. 2022), WISP2 (Frewer et al. 2013), PTN (Zhou et al. 2018), ADAMTS5 (Huang et al. 2019; Zhou et al. 2018), FMOD (Pourhanifeh et al. 2019), LUM (Chen et al. 2020)). Interestingly, for ADAMTS5, LUM, and CILP2, there is an antagonistic relationship between age‐related pathologies. ADAMTS5, for example, is an aggrecanase that degrades aggrecan, the major component of the cartilage ECM. Deletion of ADAMTS5 has been shown to prevent osteoarthritis in mouse models by preventing aggrecan degradation (Glasson et al. 2005). On the other hand, higher degradation of aggrecan has been linked to a lower risk of atherosclerosis (Z. Wang et al. 2019). This antagonistic relationship suggests a complex interplay and trade‐offs between the matrisome and age‐related diseases.

The notion that ECM protein changes are not neutral markers, but active drivers of biological aging and functional decline, was supported by their stronger associations with disease compared to other proteins (Figure 2A,B). Notably, proteins linked to the majority of chronic disease categories showed a significant enrichment for ECM components (Figure 2C). The most substantial enrichment was seen for mental and digestive diseases. The association with mental diseases was particularly intriguing: It is known that psychological stress and CNS trauma lead to biphasic remodeling of the brain ECM, marked by an initial inflammatory response and high ECM degradation (MMPs, ADAMTSs) followed by an overcompensation and overproduction of ECM during chronic states (TIMPs) (Ulbrich et al. 2021). During these remodeling steps, changes in perineural net deposition play a central role, as observed in depression, schizophrenia, progressive multiple sclerosis, and alcohol exposure (Blanco and Conant 2021; Lasek 2016; Pantazopoulos et al. 2021; H. Wang et al. 2026). Functionally, MMPs and proteases, such as neurepsin and MMP9, control synaptic plasticity and memory formation through remodeling of the perineuronal net, which restricts synaptic sprouting, and genetic mutations in such regulators are associated with a range of neurological conditions (Izumi et al. 2008; Rybakowski et al. 2009; Statzer et al. 2023; Tsilibary et al. 2014). These conserved associations between brain ECM remodeling and mental disorders could thus explain the strong association between mental disorders and ECM plasma protein abundances.

The ECM is furthermore tightly connected to immune function and remodeled during inflammation, explaining the enrichment in diseases associated with chronic inflammation (Pfisterer et al. 2021). Immune cells, for example, directly regulate the remodeling and degradation of the ECM through proteases and cytokines, and degraded ECM components can, in turn, induce inflammation (Pfisterer et al. 2021). In our findings, neoplasms are a notable exception, with proteins predicting neoplasms being depleted in ECM proteins. A possible explanation for this is that while neoplasms remodel the ECM, this remodeling is mainly limited to the immediate microenvironment of the neoplasm, with relatively few systemic changes until the later stages of disease progression.

A central question in aging research is to what degree aging mechanisms and phenotypes are shared across species. In our study, cross‐species comparison of ECM dynamics revealed pronounced age‐associated changes in matrisome proteins in both humans and mice (Figure 5A,B). However, the specific molecular signatures in the circulatory system differed between the two species (Figure 5C,D). While there is remarkable metabolic homogeneity between mice and humans, with similar molecular networks, organs, and disease pathogenesis, it has been argued previously that the molecular mechanisms of aging are distinct due to different aging rates (Demetrius 2005). On the other hand, many measurable age‐related changes on various omics levels are remarkably consistent across mammals (Lu et al. 2023; Tyshkovskiy et al. 2024; Tyshkovskiy et al. 2023). Our findings suggested that the aging ECM might be among the processes relatively distinct across species, except for secreted factors. While only 18 matrisome proteins were shared in their association with age across species, 9 of those were secreted factors, indicating that the age dynamics of secreted factors were the most conserved among the matrisome categories. We established a tight relationship between age‐related changes in the ECM and disease development; thus, one hypothesis is that on a molecular level, manifestations of chronic diseases related to the ECM differ across species. Interestingly, out of all matrisome categories, secreted factors were the least associated with human pathologies in our analysis, potentially indicating that changes in secreted factors are more conserved during aging across species because they represent age‐related changes independent of species‐specific pathologies.

One of the strongest rejuvenating interventions is heterochronic parabiosis. In line with this, we found that the ages predicted by ECM protein clocks were lower following heterochronic parabiosis (Figure 5E). Interestingly, ECM protein clocks showed elevated predicted age in all parabiosis groups compared to unsupervised models trained on all proteins, which predicted a minimal age gap in young isochronic animals (Figure S9). This suggested that the stress induced by parabiosis was specifically captured by ECM proteins, in line with it being a major surgical intervention. These proteomic findings are in line with recent work on transcriptomic aging clocks, which showed that upon heterochronic parabiosis, the ECM is the main module driving an increase in transcriptomic age (Tyshkovskiy et al. 2024). This suggests that the matrisome aging clock can capture the rejuvenating effects of heterochronic parabiosis despite the detrimental effects of the surgery and procedure mimicking aging.

In conclusion, we have characterized age‐related changes in the ECM proteome and identified striking non‐linear, U‐shaped dynamics across the lifespan. Using ECM proteins alone, we constructed accurate biological aging clocks and demonstrated that ECM proteins are more strongly associated with disease than other protein categories, with ECM age gaps further linked to disease status. While for both humans and mice, there are pronounced changes in the matrisome with age, age‐related ECM proteins show little overlap. Finally, by integrating Mendelian randomization data, we identified multiple causal ECM proteins in age‐related diseases, many of which are drug‐targetable, highlighting the ECM as a promising focus for therapeutic intervention in aging and chronic disease.

## 4 Study Limitations

This study has several unresolved questions and limitations. The underlying data used is cross‐sectional, meaning inter‐individual differences and cross‐generational cohort biases could potentially influence the results. The current models are trained on SomaScan data, which could mean that the findings are not per se generalizable to other proteomic platforms since, for example, Olink and SomaScan data correlate moderately with each other and their overlap in the screened proteome is limited (Eldjarn et al. 2023; Haslam et al. 2022). Targeted follow‐up studies could further evaluate the cross‐platform validity of these ECM components to address this limitation. Moreover, we describe a limited overlap in the mouse and human ECM proteome, which may be attributed to the fact that the aptamer‐based technology uses human sequences as targets. Although the platform is advertised to work across different species, and studies have been conducted across multiple species (Sproull et al. 2022), we cannot exclude that this, in part, influences our results. Additionally, we interpret these results from an ECM point of view. However, we also highlight that many of these proteins are part of the SASP or can be driven by inflammation, which may inflate the predictive aging signature. Nevertheless, these proteins remain capable of interacting with the ECM, and mechanistic validation studies are crucial to fully entangle their roles in ECM aging.

## 5 Materials and Methods

### 5.1 Description of Main Datasets

Blood proteomic data were obtained from three publicly available datasets. Two studies, Arthur et al. (2021) and Robbins et al. (2021), provided data on ~5000 plasma proteins. From the Arthur dataset, we extracted the proteomic data from their Table S4. For analyses, we used the healthy aging cohort (ABF300) for age‐associations and differential expression. The WU350 cohort from the same study was used in testing the stable ECM clocks and as input for classifiers. As this study provided the broadest age range, this cohort was used for the identification of effects.

The Robbins dataset consisted of the HERITAGE cohort, which is composed of adult parents and their offspring. Proteomic data and corresponding metadata were downloaded from https://motrpac‐data.org/related‐studies/heritage‐proteomics. Filtering was performed to only include individuals without missing proteomic data, leading to a final dataset of 736 individuals.

The third dataset (Lehallier et al. 2019) was downloaded from the Tables S16 and S17, which included the human and mouse plasma proteomic data and metadata, respectively.

Matrisome annotations for humans were downloaded from https://sites.google.com/uic.edu/matrisome/matrisome‐annotations/homo‐sapiens.

### 5.2 Aging Signature of Matrisome Proteins

Linear models were used to obtain the estimated effect sizes between plasma protein abundance and age, and corresponding *p*‐values. *p*‐values were subsequently adjusted using the FDR approach. Proteins were considered significant at an adjusted *p*‐value of 0.05 (*q* < 0.05). We used the following models in the respective datasets:

Arthur et al.: Protein ~ Sex + BMI + Age.

Robbins et al.: Protein ~ Sex + BMI + Race + Age.

Lehallier et al.: Protein ~ Cohort + Sex + Age.

To obtain proteins associated with the age gap in the parabiotic experiment, we applied the following model within an age group (i.e., young parabiotic or old parabiotic mice):

Lehallier et al.: ECM Age gap ~ Intervention group + Protein.

### 5.3 Non‐Linear Modeling of Protein Dynamics

To visualize non‐linear protein abundance dynamics, individual protein abundance data were scaled to *z*‐scores across individuals. Next, data was smoothed using LOESS regression using default settings from the R *stats* package (v4.4.2) with age as the predictor. Abundance over time was clustered using the ‘hclust’ function from the *stats* package using the ‘complete’ method. Given the broadest age range, clusters were defined based on visual inspection in the dataset of Arthur et al. (cut‐off height = 5). Given the similarities in platform, measured proteins, and to aid in translatability across datasets, we set the cut‐off height (cut‐off height = 7.5) to approach the same number of clusters in the dataset of Robins et al. as we observed in the dataset of Arthur et al.

### 5.4 Enrichment Analyses

Chi‐squared goodness‐of‐fit tests (Figures 2E and S5) were performed to assess enrichment or depletion of matrisome categories in Disease and Aging protein signatures relative to the full matrisome proteome using the *chisq.test* function in R. Expected frequencies were derived from the proportional representation of categories in the complete matrisome dataset. When expected frequencies were below 5 or sample sizes were below 40, a Monte Carlo simulation (2000 replicates) was used to calculate *p*‐values. Standardized residuals were calculated and interpreted as *z*‐scores to identify specific category deviations: |*z*| > 1.96 corresponds to *p* < 0.05. Positive residuals indicate enrichment; negative residuals indicate depletion.

To assess enrichment of ECM categories in disease‐ and aging‐associated matreotypes (Figures 2C and S3), we performed hypergeometric testing with our curated matrisome database as the universal background using the *phyper* function in R. *p*‐values were adjusted for multiple testing using the Benjamini–Hochberg (FDR) method.

### 5.5 Functional Enrichment Analyses

For functional interpretation of these clusters, we mined the MSigDB Hallmark, Gene Ontology, KEGG, WikiPathways, and Reactome databases using the R packages *clusterProfiler* (v4.14.4) and *ReactomePA* (v1.50.0). All measured proteins in Arthur et al. (2021) were used as a background set for enrichment analyses related to the datasets of Arthur et al. and Robbins et al., as the same proteomic platforms were used. Significant enrichments were defined at an adjusted *p*‐value (*q*‐value) cutoff < 0.10.

### 5.6 Protein‐Phenotype Associations

Protein‐phenotype association data were obtained from a publicly available dataset of 36,000 Icelandic individuals profiled using the SomaScan platform (Eldjarn et al. 2023). Only categorical and binary phenotypes were retained for analysis. Disease‐protein associations were defined at *p* < 1.0 × 10^–5^ for SMP normalized or non‐normalized analyses, as described in the original study. Disease phenotypes were manually classified according to the ICD‐10 classification system (World Health Organization 2019) (Table S7). ICD‐10 categories containing at least 10 distinct underlying phenotypes were retained; unspecific or unclassifiable phenotypes were excluded to ensure robust disease‐phenotype signatures. This yielded 8 ICD‐10 disease categories for downstream analysis.

### 5.7 Disease‐Matreotype

To derive a ECM signature broadly associated with disease (disease‐matreotype), within each ICD‐10 disease category, matrisome proteins were ranked by the number of distinct disease phenotypes with which they were associated (irrespective of directionality). Proteins meeting or exceeding the category‐specific 50th percentile of phenotype association counts were retained per category; because the threshold was defined by the minimum phenotype count at the 50th percentile rank, the proportion of proteins retained could exceed 50% where multiple proteins shared the same count at the boundary (Figure S4). Proteins were then filtered to retain only those qualifying in the top 50% across all 8 disease categories, yielding a set of matrisome proteins broadly and robustly associated with disease regardless of specific disease domain. This cross‐domain filter ensured that the final protein set reflected generalized disease relevance rather than association with any single disease category. This disease‐matreotype consists of 93 proteins (Table S8).

### 5.8 Aging‐Matreotype

For our aging‐matreotype signature, we included matrisome proteins that were previously associated with age in our analyses (FDR < 0.05) across the Arthur and Robbins data sets (Table S9) with shared directionality. This aging‐matreotype signature consisted of 100 proteins.

### 5.9 Creation of Proteomic Clocks

ECM clocks were created in a permutation fashion across 10,000 permutations. For every permutation, a random training dataset was defined from the complete dataset (Arthur = 100 individuals, Robbins = 600 individuals), and the remainder of the cohort was used as a testing set. This random sampling may lead to uneven inclusion of individuals across permutations; however, it should result in different training set composition across permutations. Next, we applied LASSO regression on this dataset (Alpha = 1, minimum lambda value as estimated after 10‐fold cross‐validation) using the R package *glmnet* (v4.1–8). For ECM clocks, only matrisome proteins were included for selection. Random clocks were generated using a Ridge regression (Alpha = 0) in the same permutation as the ECM clock to ensure the same number of predictors for equal comparison of age prediction. For unsupervised clocks, all measured proteins could be selected using a LASSO regression in a similar fashion as the ECM clocks. In both clocks, sex was taken along as a covariate. Protein abundance was log2‐normalized prior to clock creation. Training data was scaled independently of the test set to avoid data leakage, and the corresponding mean and standard deviation of the training dataset was then used to center the test data. We tested the predictive validity of these models in the remainder of the cohort by Pearson correlation between chronological and predicted ages.

A similar approach was applied for stable ECM clocks with minor differences. Stable ECM clocks were trained on the full dataset with the alpha parameter set to 0 to conduct a Ridge regression. Sex was excluded as a covariate in these models.

### 5.10 Age Gaps

To obtain unbiased estimates of biological age‐gaps in the studies of Arthur & Robbins, we corrected original age gaps (Predicted age—Chronological age) as suggested by de Lange and Cole (2020). In short, after age prediction, we fitted a novel linear model (“lm‐correction”) to estimate the predicted age from the chronological age. Using this model, we applied the correction as follows:

Unbiased Age Prediction = (Predicted Age−Intercept of lm‐correction)/Coefficient of lm‐correction. For age predictions in other datasets, no correction was applied to test the off‐the‐shelf validity of the models on log2 and scaled protein abundance inputs.

### 5.11 Receiver Operating Characteristic Analysis

Areas under the curve values (AUC) were estimated using the cv.glmnet function from the R *glmnet* package, using the “binomial” family as an input argument. Input data consisted of a binarized Case–Control identifier as the dependent variable and scaled abundance levels of all selected matrisome proteins (14 or 8 protein signature) as predictors. Displayed AUCs were calculated based on aggregated leave‐one‐out cross‐validation predictions.

### 5.12 Mendelian Randomization

To prioritize matrisome proteins for therapeutic interventions, we leveraged the results of a phenome‐wide Mendelian Randomization study (Su et al. 2024). Results were obtained from https://broad.io/protein_mr_atlas. In short, this study conducted Mendelian randomization in three ancestries (European, African, East‐Asian) across seven cohorts. We extracted all significant matrisome proteins (Wald ratio or Inverse variance weighted method, *p* < 0.05) on the SomaScan platform.

### 5.13 Protein‐Drug Interactions

Drug targets were identified using the Human Protein Atlas (v24; https://www.proteinatlas.org/about/download) (Jiang et al. 2020) and the Drug Gene Interaction database (https://dgidb.org/downloads; version 2024‐Dec) (Cannon et al. 2024). The latter was also used to identify drug‐gene interactions. Databases were filtered on the gene symbols of matrisome proteins.

Protein‐drug and protein–protein interactions were identified using the STRING database (v12), accessed through the R package STRINGdb (v2.22.0). A high threshold (score = 700) was used to identify both functional and physical protein–protein interactions. These interactions were combined with protein‐drug interactions to obtain the final protein‐drug interaction network.

## Author Contributions

C.Y.E., L.C., and J.M. designed the study. L.C. and A.M. performed the analyses. All authors participated in interpreting the data. L.C., C.Y.E., and A.M. wrote the manuscript in consultation with the other authors.

## Funding

This work was supported by Funding from the Swiss National Science Foundation Funding from the SNF P3 Project 190072 to C.Y.E. and Alzheimer Nederland (WE.03‐2020‐13) granted to J.M.

## Conflicts of Interest

The authors declare no conflicts of interest. With no relation to the present manuscript, C.Y.E. declares to be a co‐founder and shareholder of Avea Life AG and Lichi3 GmbH. C.Y.E. and A.M. are employed by Novartis. B.L. is a full‐time employee of Alkahest Inc. Correspondence should be addressed to J.M. and C.Y.E.

## Supporting information


**Data S1:** Supplementary Tables S1–24

**Figure S1:** Validation of age‐associated matrisome changes. (A) Overview of the number of matrisome proteins (MPs) per matrisome category in the dataset of Robbins et al. 2021. (B) Results of the linear modeling in Robbins et al. (2021), with the specified model on top. *x*‐axis denotes the effect size, and *y*‐axis denotes the −log10(FDR) value. Labeled proteins are highlighted based on functional description in the text. (C) Relative expression trajectories of *z*‐scored protein abundances across samples per category of MP over time. (D) Zoom in on a single category of MPs, including its underlying proteins. Thick lines reflect the mean expression trajectory, and thin lines denote *z*‐scored protein abundances across the trajectories of individual proteins. (E) Cnetplot denoting the enriched function/process in the bigger circles, with the connected proteins in each small circle. Colors denote in which cluster the function or protein is found.

**Figure S2:** SASP not a driver of age‐associated matrisome changes. Mean age‐associated normalized expression trajectories of SASP (blue) and non‐SASP (red) proteins within each matrisome category.

**Figure S3:** Disease enrichment across ECM categories. ICD‐10 disease chapter enrichment for individual matrisome categories. Shown is the log2 fold change of enrichment or depletion of all ICD10 disease chapters for each category of ECM proteins. Color indicates −log10 Bonferroni‐adjusted *p*‐value. Gray color indicates the *p*‐value did not reach the threshold of *p* < 0.05.

**Figure S4:** Density distributions of phenotype associations. Depicted are the densities of phenotype associations for the proteins of the highlighted ICD‐10 disease chapters. Highlighted area indicates proteins above the threshold for selection of proteins with high disease association (top 50% of proteins).

**Figure S5:** Matrisome category enrichment analysis in disease and aging protein signatures. Standardized residuals from chi‐squared goodness‐of‐fit tests comparing the distribution of matrisome categories in Disease (gold) and Aging (blue) protein signatures against the expected distribution from the full matrisome. Positive residuals indicate over‐representation (enrichment) and negative residuals indicate under‐representation (depletion) of specific categories. Vertical dashed lines mark statistical significance thresholds at ±1.96 (*p* < 0.05). Bars with full opacity represent statistically significant deviations (|z| > 1.96); faded bars indicate non‐significant differences.

**Figure S6:** Aging and disease signature trajectories in the Robbins dataset. (A) Smoothed and normalized mean protein expression trajectories are shown as a function of chronological age in the Robbins dataset (blue: aging signature, gold: disease signature, gray: all matrisome proteins). 95% confidence intervals are indicated for each line. (B) Similar to (A), but stratified by ECM category (ECM glycoproteins, collagens, proteoglycans, ECM‐affiliated proteins, ECM regulators, and secreted factors).

**Figure S7:** Predictive validity of the 14‐protein ECM clock. Overview of predictive validity of stable 14 protein ECM clock versus 14 random protein clocks (A, B) or 8 protein ECM clock versus 8 random protein clocks (C, D) across 10,000 permutations in the two testing datasets. (E) Application of the reduced 8 protein models to the datasets previously used for testing the 14 ECM protein models. Correlational strength represents Spearman correlations. Age gap difference and correspondence significance level are estimated by the Wilcoxon tests, with the ‘control’ groups as reference. AUC values indicate the aggregated AUC of the model in this dataset using a leave‐one‐out cross‐validation. **p < 0.01, *p < 0.05, # *p* < 0.10.

**Figure S8:** Correlation matrix of age gaps with Alzheimer's disease. Correlational plot reflecting significant associations (uncorrected *p* < 0.05) between the age gap observed in the Dammer et al. (2022) study (‘ageGapRobbins’) and clinical metadata associated with Alzheimer's disease.

**Figure S9:** Age‐gaps predicted from the ECM clock in the parabiosis experiment. Comparison of age‐gaps across parabiotic groups estimated using random protein aging clocks trained in healthy, non‐parabiotic mice.

## Data Availability

The data that supports the findings of this study are available in the Supporting Information of this article.
