# Supplementary figures and images for: An Extracellular Matrix Aging Clock Based on Circulating Matrisome Proteins Predicts Biological Aging and Disease
Source: Aging Cell. 2026 Apr 15;25(4):e70474. doi: 10.1111/acel.70474 (PMC13083228; doi:10.1111/acel.70474)

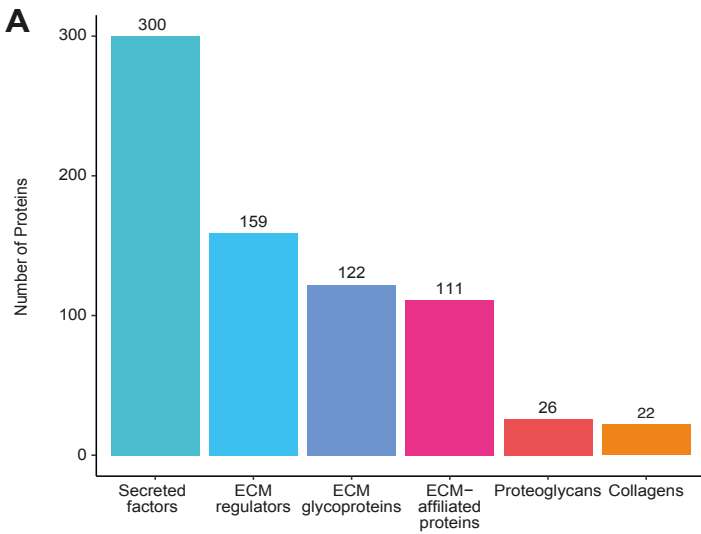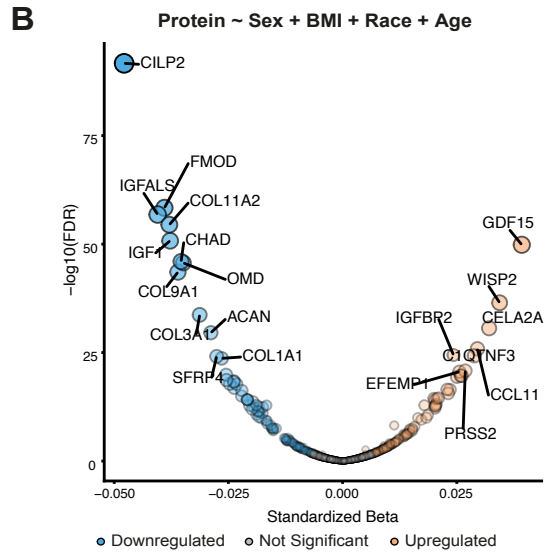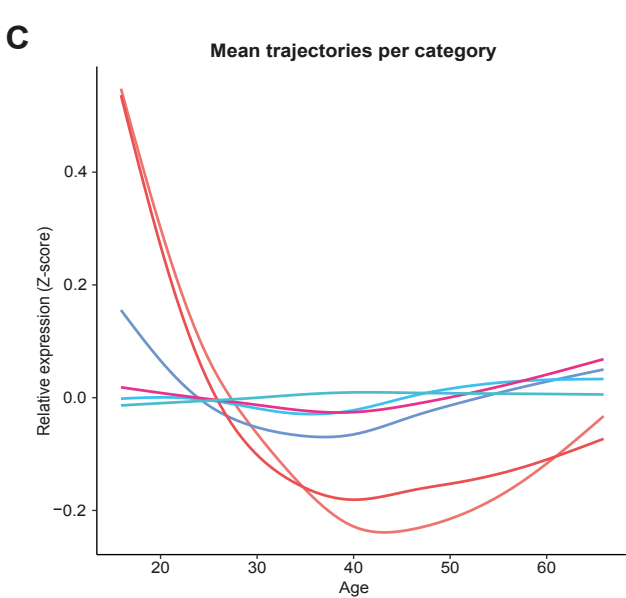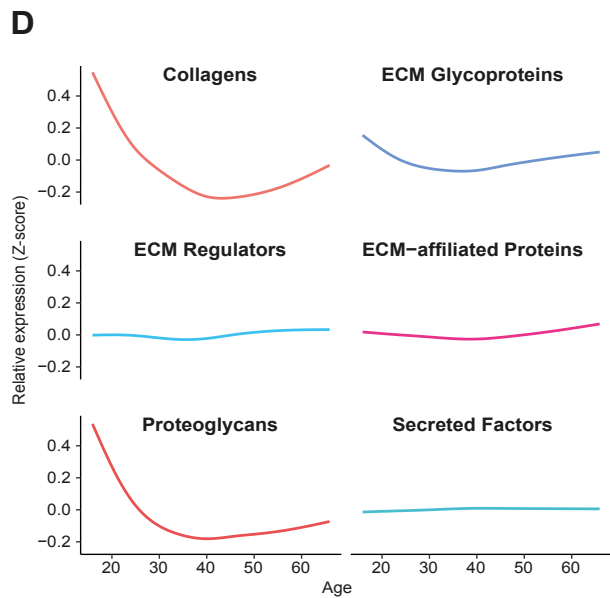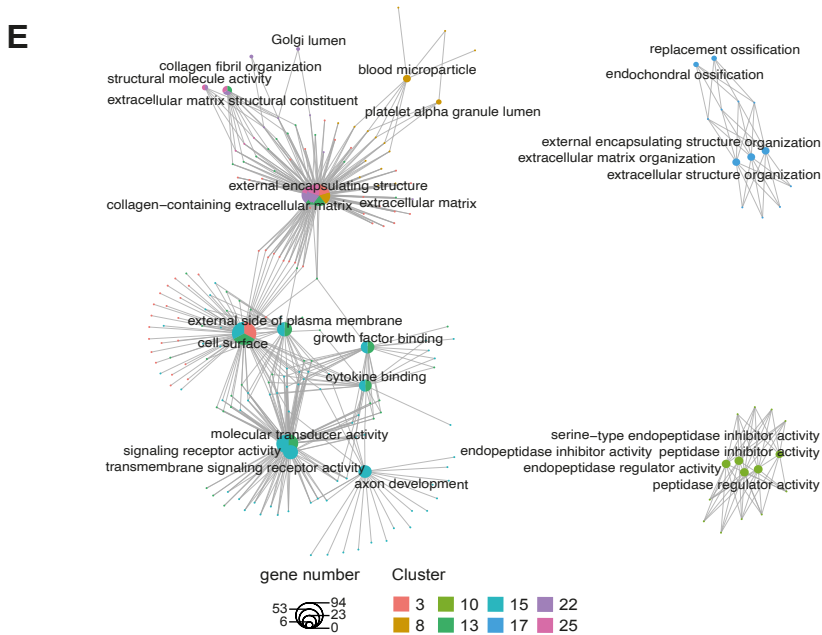

Supplement: Supplementary file 2 — Figure S1: Validation of age‐associated matrisome changes. (A) Overview of the number of matrisome proteins (MPs) per matrisome category in the dataset of Robbins et al. 2021. (B) Results of the linear modeling in Robbins et al. (2021), with the specified model on top. x‐axis denotes the effect size, and y‐axis denotes the −log10(FDR) value. Labeled proteins are highlighted based on functional description in the text. (C) Relative expression trajectories of z‐scored protein abundances across samples per category of MP over time. (D) Zoom in on a single category of MPs, including its underlying proteins. Thick lines reflect the mean expression trajectory, and thin lines denote z‐scored protein abundances across the trajectories of individual proteins. (E) Cnetplot denoting the enriched function/process in the bigger circles, with the connected proteins in each small circle. Colors denote in which cluster the function or protein is found. [file ACEL-25-e70474-s009.pdf]

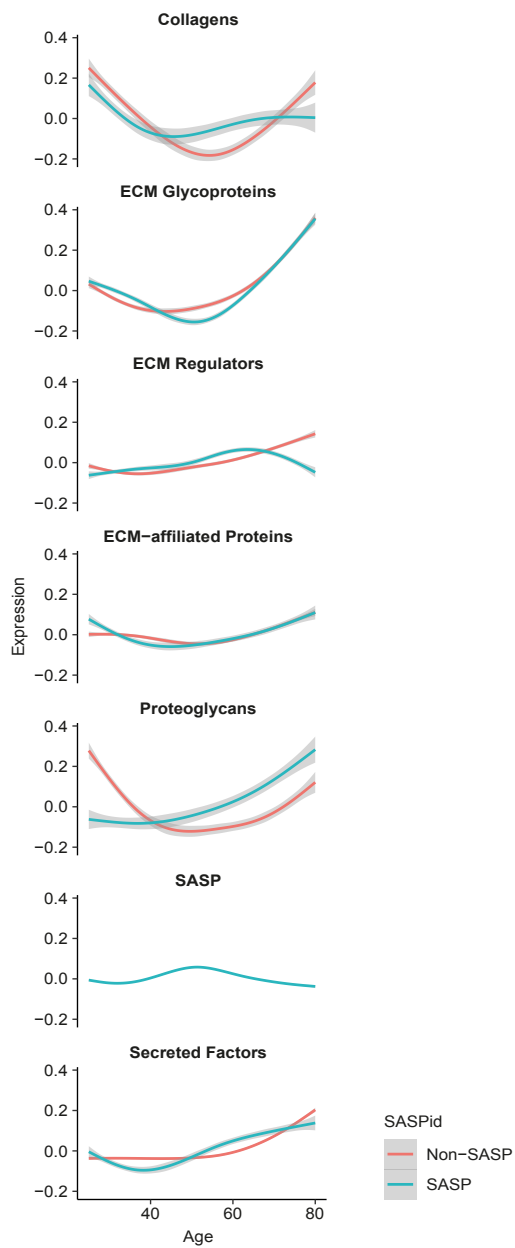

Supplement: Supplementary file 3 — Figure S2: SASP not a driver of age‐associated matrisome changes. Mean age‐associated normalized expression trajectories of SASP (blue) and non‐SASP (red) proteins within each matrisome category. [file ACEL-25-e70474-s003.pdf]

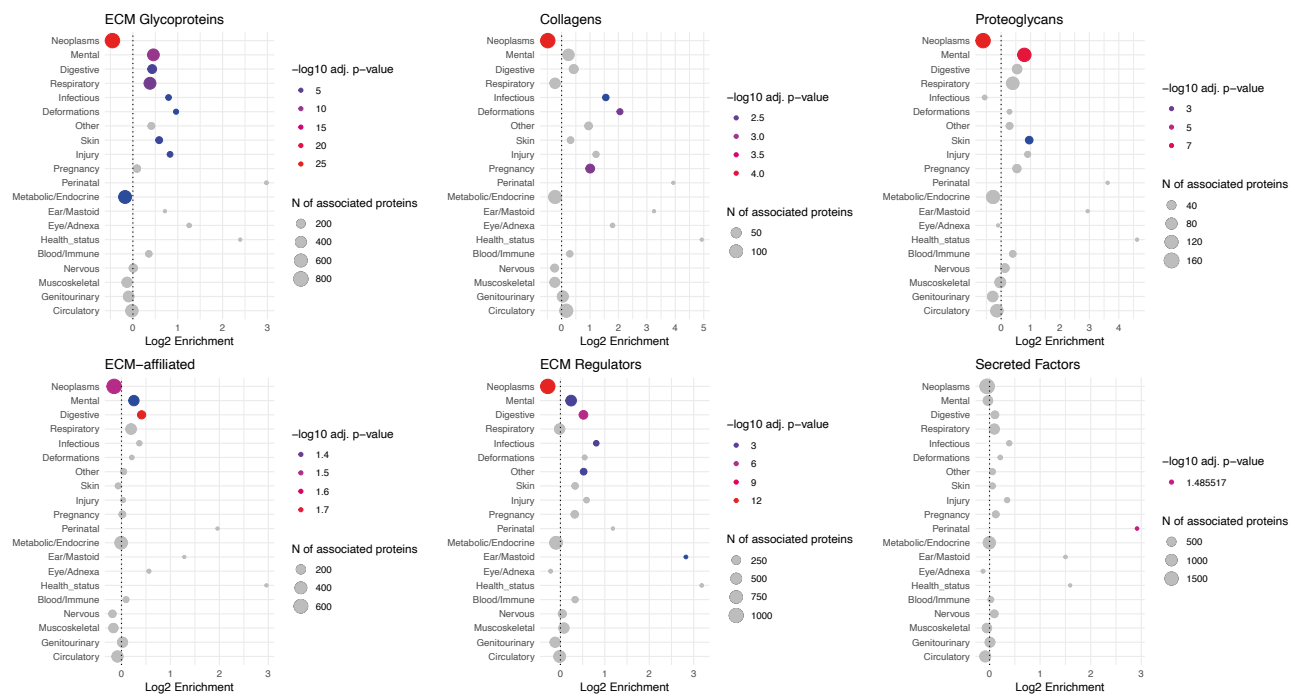

Supplement: Supplementary file 4 — Figure S3: Disease enrichment across ECM categories. ICD‐10 disease chapter enrichment for individual matrisome categories. Shown is the log2 fold change of enrichment or depletion of all ICD10 disease chapters for each category of ECM proteins. Color indicates −log10 Bonferroni‐adjusted p‐value. Gray color indicates the p‐value did not reach the threshold of p < 0.05. [file ACEL-25-e70474-s008.pdf]

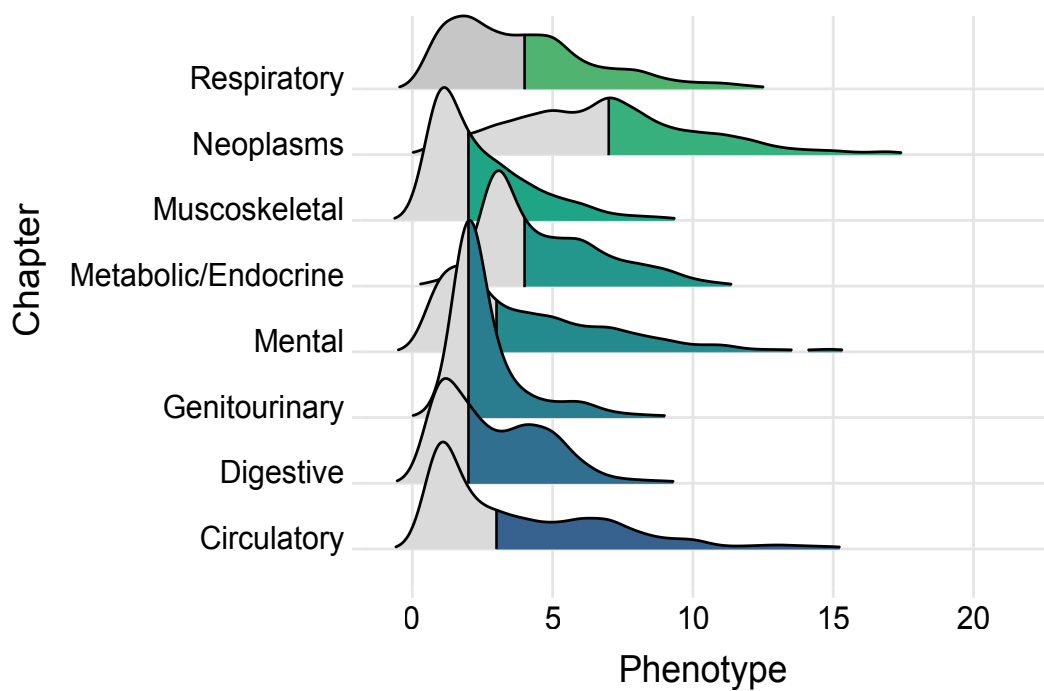

Supplement: Supplementary file 5 — Figure S4: Density distributions of phenotype associations. Depicted are the densities of phenotype associations for the proteins of the highlighted ICD‐10 disease chapters. Highlighted area indicates proteins above the threshold for selection of proteins with high disease association (top 50% of proteins). [file ACEL-25-e70474-s007.pdf]

Signature   Aging   Disease

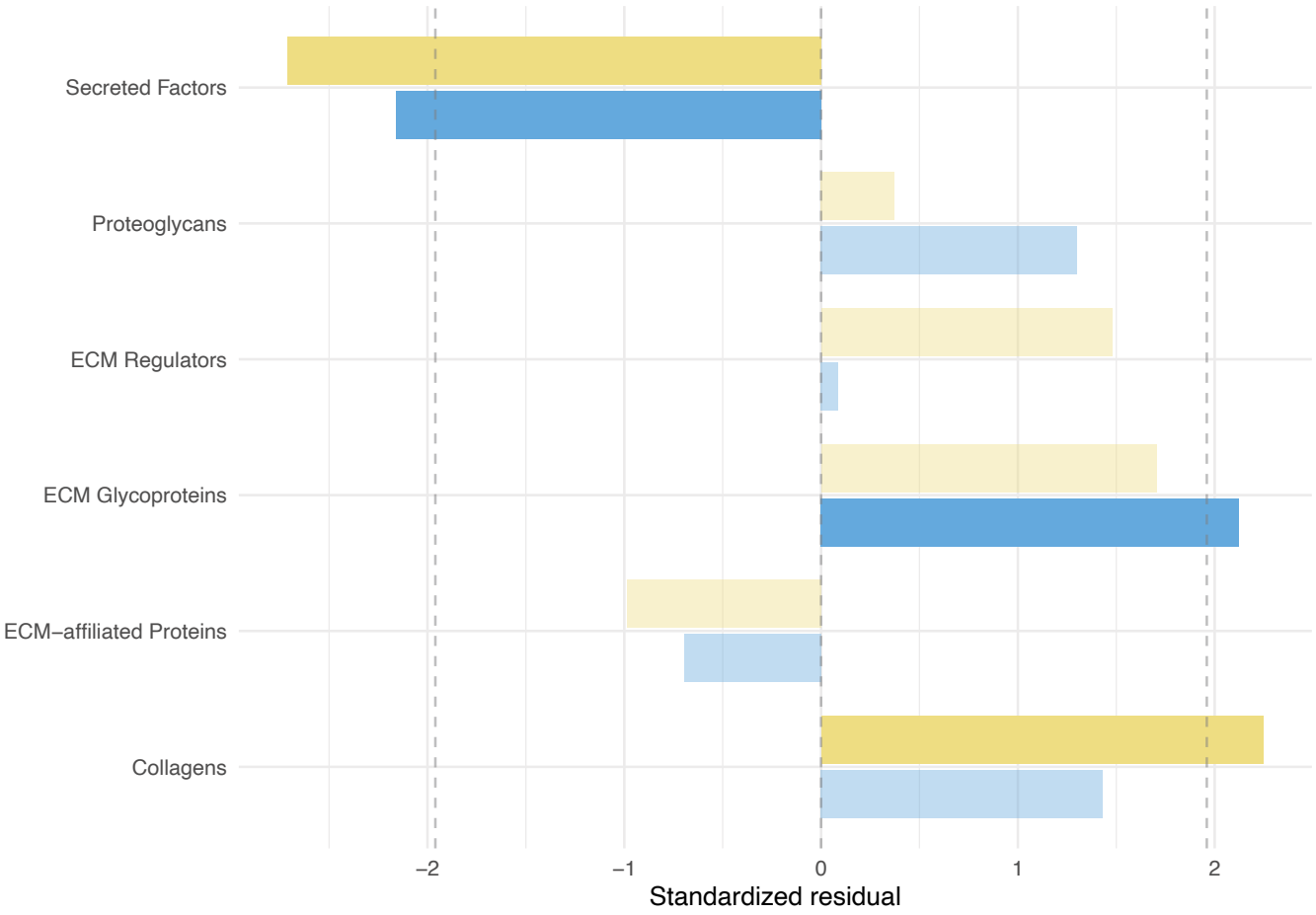

Supplement: Supplementary file 6 — Figure S5: Matrisome category enrichment analysis in disease and aging protein signatures. Standardized residuals from chi‐squared goodness‐of‐fit tests comparing the distribution of matrisome categories in Disease (gold) and Aging (blue) protein signatures against the expected distribution from the full matrisome. Positive residuals indicate over‐representation (enrichment) and negative residuals indicate under‐representation (depletion) of specific categories. Vertical dashed lines mark statistical significance thresholds at ±1.96 (p < 0.05). Bars with full opacity represent statistically significant deviations (|z| > 1.96); faded bars indicate non‐significant differences. [file ACEL-25-e70474-s002.pdf]

**A**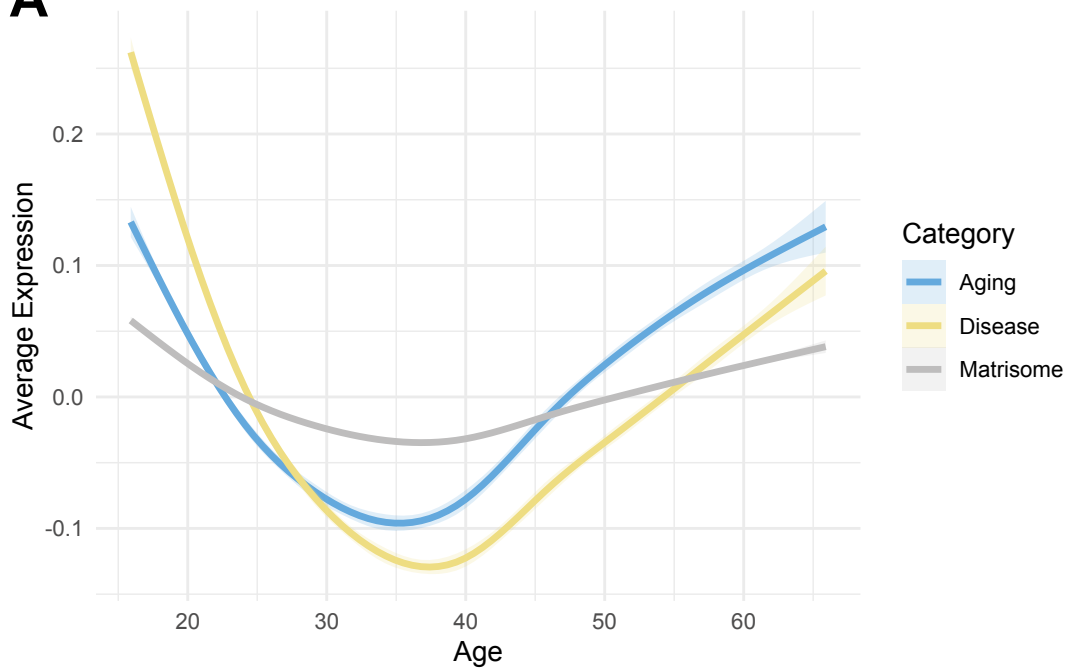**B**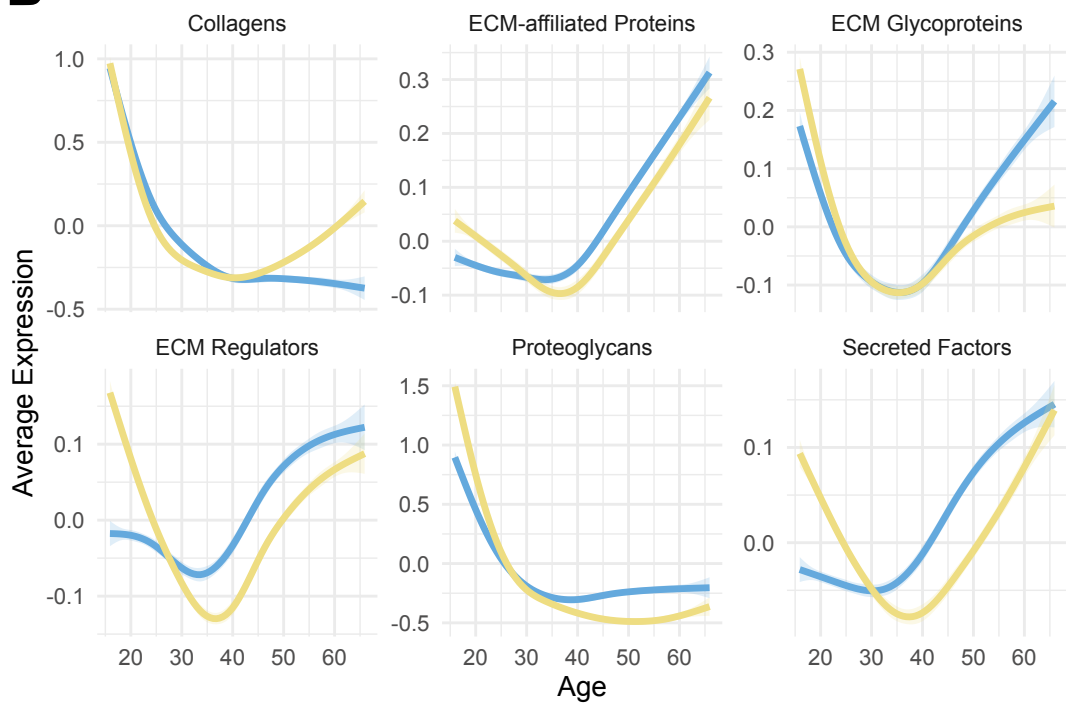

Supplement: Supplementary file 7 — Figure S6: Aging and disease signature trajectories in the Robbins dataset. (A) Smoothed and normalized mean protein expression trajectories are shown as a function of chronological age in the Robbins dataset (blue: aging signature, gold: disease signature, gray: all matrisome proteins). 95% confidence intervals are indicated for each line. (B) Similar to (A), but stratified by ECM category (ECM glycoproteins, collagens, proteoglycans, ECM‐affiliated proteins, ECM regulators, and secreted factors). [file ACEL-25-e70474-s001.pdf]

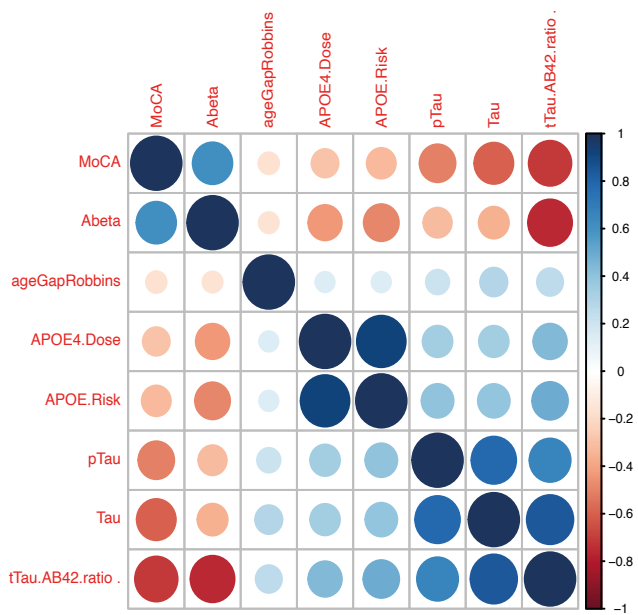

Supplement: Supplementary file 9 — Figure S8: Correlation matrix of age gaps with Alzheimer's disease. Correlational plot reflecting significant associations (uncorrected p < 0.05) between the age gap observed in the Dammer et al. (2022) study (‘ageGapRobbins’) and clinical metadata associated with Alzheimer's disease. [file ACEL-25-e70474-s006.pdf]

**A****Parabiosis – Unsupervised clocks**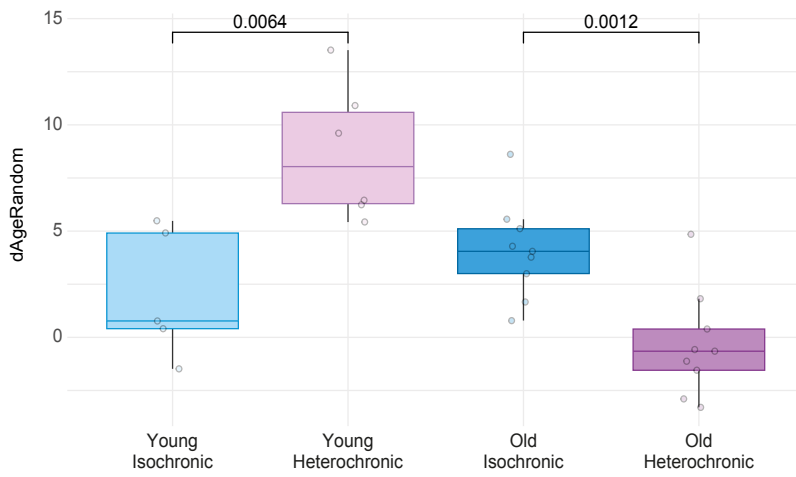

Supplement: Supplementary file 10 — Figure S9: Age‐gaps predicted from the ECM clock in the parabiosis experiment. Comparison of age‐gaps across parabiotic groups estimated using random protein aging clocks trained in healthy, non‐parabiotic mice. [file ACEL-25-e70474-s010.pdf]
