# Supplementary material for: An Extracellular Matrix Aging Clock Based on Circulating Matrisome Proteins Predicts Biological Aging and Disease
Source: Aging Cell. 2026 Apr 15;25(4):e70474. doi: 10.1111/acel.70474 (PMC13083228; doi:10.1111/acel.70474)
Supplement: Supplementary file 8 — Figure S7: Predictive validity of the 14‐protein ECM clock. Overview of predictive validity of stable 14 protein ECM clock versus 14 random protein clocks (A, B) or 8 protein ECM clock versus 8 random protein clocks (C, D) across 10,000 permutations in the two testing datasets. (E) Application of the reduced 8 protein models to the datasets previously used for testing the 14 ECM protein models. Correlational strength represents Spearman correlations. Age gap difference and correspondence significance level are estimated by the Wilcoxon tests, with the ‘control’ groups as reference. AUC values indicate the aggregated AUC of the model in this dataset using a leave‐one‐out cross‐validation. **p < 0.01, *p < 0.05, # p < 0.10. [file ACEL-25-e70474-s005.pdf]

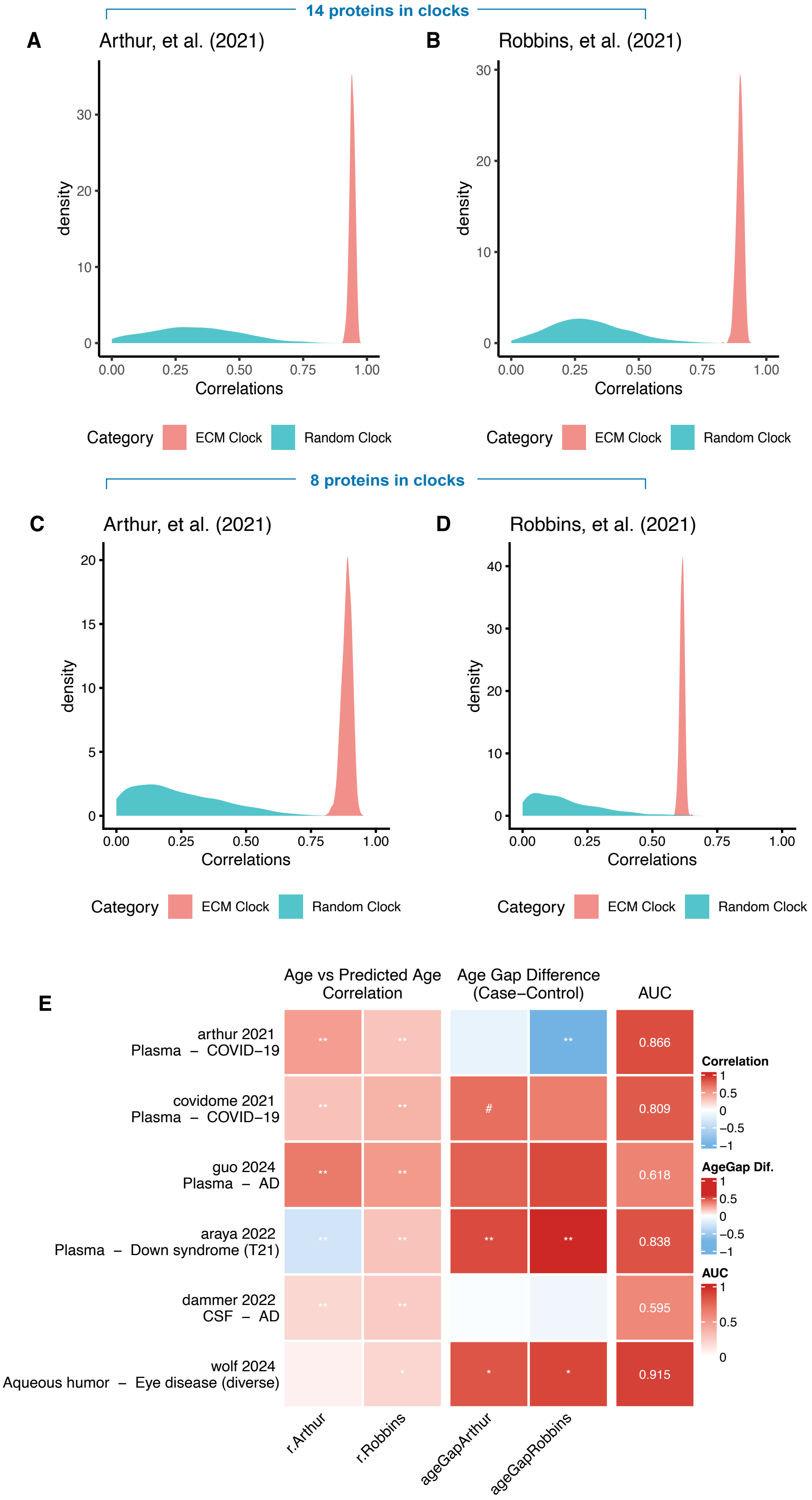

14 proteins in clocks
A
Arthur, et al. (2021)
B
Robbins, et al. (2021)
density
Correlations
Category
ECM Clock
Random Clock
8 proteins in clocks
C
Arthur, et al. (2021)
D
Robbins, et al. (2021)
E
Age vs Predicted Age Correlation
Age Gap Difference (Case−Control)
AUC
arthur 2021 Plasma − COVID−19
covidome 2021 Plasma − COVID−19
guo 2024 Plasma − AD
araya 2022 Plasma − Down syndrome (T21)
dammer 2022 CSF − AD
wolf 2024 Aqueous humor − Eye disease (diverse)
0.866
0.809
0.618
0.838
0.595
0.915
r.Arthur
r.Robbins
ageGapArthur
ageGapRobbins
Correlation
AgeGap Dif.
AUC
